# A modified TOP assay to detect per- and polyfluoroalkyl substances in aqueous film-forming foams (AFFF) and soil

**DOI:** 10.3389/fchem.2023.1141182

**Published:** 2023-10-10

**Authors:** Md. Al Amin, Yunlong Luo, Feng Shi, Linbo Yu, Yanju Liu, Annette Nolan, Olalekan Simon Awoyemi, Mallavarapu Megharaj, Ravi Naidu, Cheng Fang

**Affiliations:** ^1^ Global Centre for Environmental Remediation (GCER), University of Newcastle, Callaghan, NSW, Australia; ^2^ CRC for Contamination Assessment and Remediation of the Environment (CRC CARE), University of Newcastle, Callaghan, NSW, Australia; ^3^ Ramboll Australia, The Junction, NSW, Australia

**Keywords:** TOP assay, PFAS precursors, AFFF, fluorotelomer sulfonate (FTS), solid-phase extraction (SPE), oxidation efficiency

## Abstract

Total oxidisable precursor (TOP) assay can oxidise some per- and polyfluoroalkyl substances (PFASs) and their precursors, most of which cannot be quantitatively detected so far, and convert them to detectable PFASs, such as perfluoroalkyl acids (PFAAs). However, the conversion is constrained by the complexity of the target samples, including co-existent organics, unknown PFAS precursors, and background. In this study, the TOP assay is modified to increase the oxidation and conversion efficiency by changing the initial concentration of target sample, increasing oxidising doses, time, temperature, etc. The modified TOP assay is applied to test several aqueous film-forming foams (AFFF) and a PFAS-contaminated soil extract. The sum concentrations of the detectable PFASs are increased by up to ∼534× in the AFFF samples and ∼7× in the PFAS-contaminated soil extract. The detectable fluorotelomer sulfonate (FTS, such as 6:2/8:2 FTS) is accounted as an oxidation indicator to monitor the oxidation and conversion progress of the oxidisable PFASs precursors to the detectable PFASs. Overall, the modified TOP assay could be an appropriate method for identifying missing PFASs mass in complex matrices by detecting the PFASs precursors effectively.

## 1 Introduction

Per- and polyfluoroalkyl substances (PFASs) have been widely used due to their unique properties, such as the simultaneous oleophobicity and hydrophobicity as surfactants (Buck et al., 2011). The carbon-fluorine (C-F) skeleton is highly resistant to most forms of oxidation; hence the chemical or biological degradation is often difficult (Wang et al., 2014b; Trojanowicz et al., 2018). On the other hand, PFAS precursors are C-F-containing structures (i.e., PFAS) with transformable moieties (of non-C-F). These kinds of structures can still result in terminal PFAS end products after transformation or conversion upon chemical or thermal treatment to remove the non-C-F moieties. So far, nearly 5,000 PFASs, including precursors, have been estimated to exist in the PFASs global market (OECD, 2018; Zhang et al., 2019). Unfortunately, only a limited number of PFASs can be quantitatively detected. Even within this limitation, there has been widespread of PFASs that have been globally detected in environmental and biological matrices, such as water, soil, sediment, foodstuff, air, dust, wildlife species and human blood serum (Yao et al., 2018; Chen et al., 2019; Zhu and Kannan, 2020).

As an application example, aqueous film-forming foams (AFFF) have been previously formulated with PFASs (Place and Field, 2012; Turner et al., 2021) to combat and extinguish combustible and flammable liquid fuel-caused fires (Bolan et al., 2021). Because of their frequent use (for fire training practice, for example) at firefighting brigades, ships, airports, and military bases over previous decades (Barzen-Hanson et al., 2017; Nason et al., 2020; Turner et al., 2021), AFFF have become a significant source of the PFASs contamination. However, due to commercialisation reasons, the exact formulation of AFFF is generally unknown (Mejia-Avendaño et al., 2017), which makes it complicated for PFASs detection. The thermodynamic and kinetics of conversion of the PFASs precursors to the end products and the detectable PFASs (as intermediates) further obscure the AFFF detection.

Once entered the environment and for instance leaked into soil, PFASs can be retained in the soil by partition, sorption, and complexation reactions (for example, with the involvement of biotas or bio-conversion of the precursors) (Bolan et al., 2021). Consequently, PFAS-contaminated soil can become a source of PFASs contamination, which can be further transported by leaching from soil to surface water, groundwater and biotas (Xiao et al., 2015; Turner et al., 2021). While the detection of perfluoroalkyl acids (PFAAs), such as perfluoroalkyl carboxylic acids (PFCA) and perfluoroalkyl sulfonic acids (PFSA) in soil has been well documented (Houtz et al., 2013; Janda et al., 2019; Nickerson et al., 2020), consideration of the PFAS precursors in the environment is still required (Martin et al., 2019). However, detection of PFASs including precursors in the soil matrix is generally more complicated than that in the water phase.

To date, high-performance liquid chromatography-tandem mass spectrometry (HPLC-MS/MS) is the most widely used technique for detecting PFASs and their end products (Janda et al., 2019; Al Amin et al., 2020). However, identifying PFAS in AFFF or the AFFF impacted soil is still challenging. The possible reasons include i) certain PFASs that cannot be quantified analytically because of the limited number of standards; ii) complex structure of the PFASs formulation itself, including precursors and co-existence; iii) thermodynamic and kinetic conversion from precursors after entering the environment, due to the breakdown of the non-C-F moieties (partially or fully); and iv) the extra complexity of the soil matrices owing to the presence of organic and inorganic compounds.

In order to detect the PFASs including precursors in water and soil matrices, different methods have been developed, such as extractable organic fluorine (EOF) (Yeung et al., 2013; Codling et al., 2014; Tan et al., 2014), absorbable organic fluorine (AOF) (Wagner et al., 2013; Willach et al., 2016; Bach et al., 2017), and the total oxidisable precursor (TOP) assay (Houtz et al., 2013; Houtz et al., 2016; Göckener et al., 2021; Göckener et al., 2022). Among those methods, the TOP assay has been shown to be very specific to the analysis of the PFASs precursors (Houtz and Sedlak, 2012; Martin et al., 2019; Gockener et al., 2020; Gockener et al., 2021). Consequently, the TOP assay has been advanced significantly (Slee et al., 2019; Göckener et al., 2021).

The TOP assay was first introduced by Houtz and Sedlak (2012). Ideally, during the oxidative treatment, the PFASs precursors that contain non-C-F moieties are selectively oxidised and converted primarily to detectable PFCA products or other PFASs end products. However, the complete and selective oxidation of non-C-F moieties requires aggressive reaction conditions (Mejia-Avendaño et al., 2017), depends on the sample’s background and complexity, and the TOP assay parameters (Slee et al., 2019). For an incomplete oxidation, the TOP assay might yield some intermediates, which makes the TOP assay results very challenging and difficult to interpret (Göckener et al., 2021). In other words, the TOP assay should be modified and applied for different kind of matrices (Houtz et al., 2013; Dauchy et al., 2017; Janda et al., 2019; Slee et al., 2019; Zhang et al., 2019; Gockener et al., 2020; Hutchinson et al., 2020; Rodowa et al., 2020; Göckener et al., 2021). Recently, the TOP assay was applied by adding additional amount of oxidative agent to test AFFF samples (Al Amin et al., 2021). Whether or not the modified TOP assay can be applied for the soil testing is still being investigated.

In this study, the TOP assay is further modified and enhanced to detect the PFASs and precursors in AFFF samples with different initial concentrations and to analyse the PFASs in soil. While the detectable and oxidisable fluorotelomer sulfonate (FTS, such as 6:2/8:2 FTS) can be employed as an indicator to monitor the oxidation process, the drawbacks and other concerns on the TOP assay are also discussed. Using the indicator molecule, we can approach the complete oxidation by diluting sample, enlarging the oxidative load, increasing the temperature, and prolonging the reaction times. After being modified, TOP assay is validated and applied for soil test. The results are helpful for PFAS test that is still a challenge so far.

## 2 Materials and methods

### 2.1 Standards and reagents

All the PFASs standards were obtained from National Measurement Institute (NMI), Australia. HPLC-MS grade methanol, water, ammonium hydroxide, ammonium acetate, perfluorooctanoic acid (PFOA), perfluorooctane sulfonate potassium salt (PFOS), potassium persulphate (K_2_S_2_O_8_), sodium hydroxide (NaOH) and hydrochloric acid (HCl) were procured from Sigma-Aldrich (Australia). Supelclean ENVI-Carb from Supelco was used for solid-phase extraction (SPE), with Supelclean™ ENVI-WAX. Glassware was intentionally avoided, and low-density polypropylene (PP) test tubes and pipette tips were used to conduct all experiments. Ultrapure water was used for dilution and cleaning purposes.

A list of analysed PFASs and internal standards in this study are presented in Supplementary Table S1 (Supporting Information).

### 2.2 Samples collection and extraction

#### 2.2.1 AFFF samples

Four different AFFF samples obtained from the Department of Defence (Australia) were tested in this study, including Orchidee (#1), Wormald (#2), and Ansulite (#3 and #4). All the samples were transported to the University of Newcastle in a cooler box and stored at room temperature before conducting any experiment. However, all AFFF samples were appropriately shaken and then transferred into the PP centrifuge tube after substantial foaming and stratification became static. To dilute the AFFF samples at 5,00,000×, 20,000×, 1,000× and 100× (in volume) into 50 mL PP tubes with milli-Q water (MQ water, >18 MΩ·cm at 25°C), a segment of serial dilution was performed to avoid inaccuracy of the bulk concentration. In the meantime, for the proper recovery of PFASs, a little amount of methanol (<1%) was added.

#### 2.2.2 Soil sample

PFAS-contaminated soil was obtained from Germany (directly sent to the University of Newcastle, Australia). Once received, the soil was dried overnight and sieved with a 2.0-mm sieve (Lei et al., 2020). After that, the soil was stored at room temperature. Before the test, the pre-treated soil was extracted according to previous studies, with minor modification (Rich et al., 2015; Zhu and Kannan, 2020). Briefly, i) ∼1.0 g of soil was weighed into a 15 mL PP tube; ii) 10 mL of 0.1% ammonium hydroxide in methanol was added; iii) sample was sonicated at ∼35°C for ∼30 min, followed by mixing and shaking at 250 rpm for ∼2 h, to maximise the recovery of target; iv) liquid phase was transferred into another 50 mL new PP tube after centrifugation at 5,000 rpm for ∼10 min, and steps [(ii) to (iv)] were repeated twice. Subsequently, all the extracts were combined and concentrated to 1 mL using a gentle stream of nitrogen at ∼40°C.

The soil extract was divided into two equal aliquots (one as before, and another for the TOP assay). Extract was then diluted using MQ water at 10×, 50× or 100× into 5 mL in a 15 mL PP tube, and then subject to the TOP assay.

### 2.3 Modified TOP assay

The TOP assay protocol was followed according to (Houtz and Sedlak, 2012), and was modified for both the AFFF samples and the soil extract. Briefly, the initial concentration of the TOP assay reagents 0.06 M K_2_S_2_O_8_ (concentration of 1×) and 0.15 M NaOH (concentration of 1×) (Houtz and Sedlak, 2012) were increased to 0.48 M (concentration of 8×, when compared to 0.06 M of 1×) and 1.5 M (concentration of 10×, when compared to 0.15 M of 1×), respectively, and added to a ∼5 mL volume containing AFFF samples or the soil extract in polypropylene tubes and mixed properly. After that, all AFFF and soil samples were put in a water bath at ∼90°C (or others as indicated below) for ∼7 h and shaken manually after a certain time interval (∼30 min). The samples were then cooled down to the room temperature (∼24°C), and pH was adjusted with concentrated HCl to pH 5–9 to stop the further oxidation reaction. Finally, all the samples were kept at ∼4ºC for solid phase extraction (SPE) prior to HPLC-MS/MS analysis.

Prior to the HPLC-MS/MS analysis, as a clean-up and concentration step, SPE was conducted using Supelclean™ ENVI-WAX. The SPE cartridges were pre-washed with ∼3 mL of 0.1% ammonia in methanol, 3 mL of methanol and pre-conditioned with 3 mL of HPLC-MS grade water twice. After the cartridges were rinsed with ∼3 mL of HPLC-MS grade water twice, the cartridges were dried under vacuum. Elution of analytes was performed with ∼3 mL of methanol and ∼7 mL of acetonitrile (ACN) for AFFF, while ∼1.5 mL methanol and ∼3.5 mL ACN were used for soil extract. The samples were pulled through the cartridges at a speed of approximately 1 drop/second. The combined eluates (∼10 mL for AFFF, and ∼5 mL for soil sample) were dried under a gentle stream of nitrogen at ∼40°C to keep the volume at ∼0.5 mL. Vortex and ultra-centrifugation were performed for proper homogenisation to avoid any precipitation. Further details on the TOP assay are shown in Supplementary Figures S1, S2 (Supplementary Material). The modified TOP assay might be applied to other environmental samples, as shown in Supplementary Figures S1, S3 (Supplementary Material).

All samples and extracts were filtered with a 0.22 μm cellulose acetate syringe filter and the internal standards were added into each sample (details in Supplementary Table S1, Supplementary Material), prior to the HPLC-MS/MS analysis. The solutions were then transferred into HPLC vials before loading the tray in the HPLC system, as described in the next section.

### 2.4 PFASs analysis

For the PFASs analysis, the standard method (EPA/600/R-08/092) was used to conduct HPLC-MS/MS (Shoemaker, 2009). Briefly, a volume of 10 µL was injected into an Agilent 1260 high-performance liquid chromatography system (Agilent, CA, United States) fitted with an Eclipse Plus-C18 column (internal diameter: 4.6 mm, length: 100 mm, and particle size: 3.5 mm), which was heated to ∼40°C. Mixtures of ammonium acetate (10 mM): water (10:90, v/v) were used for mobile phase A, methanol (100%, v/v) for mobile phase B, and sonication was used to degas both mobile phases. A constant flow rate of 0.4 mL/min was set for the gradient elution. The nebuliser gas (nitrogen) pressure was set at 35 psi, the drying gas flow rate was 10 mL/min, the temperature was set to 350°C, and the capillary voltage was +3,500 V (Vecitis et al., 2008). A Quadrupole 6470 (triple quadrupole mass spectrometer) detector was operated in the negative electro-spray ionization (ESI) mode using multiple reaction monitoring (MRM) (Supplementary Table S1) for scanning specific target analytes in the analysed samples. For example, PFOS and PFOA were quantified with multiple reaction monitoring (MRM) employing the most abundant precursor ion transitions (499 to 79.9 for PFOS and 413 to 369 for PFOA).

### 2.5 Quality assurance (QA)/quality control (QC)

For QA/QC, at least three samples were run in parallel for each batch of the test (Wang et al., 2014a), including one for the “pre TOP assay,” one for the “post-TOP assay,” and one for the “calibration test” (Fang et al., 2018). Methanol and water (HPLC-MS grade) were used to prepare the samples including blanks, which were run before and after each set of tests to minimise the background contamination. 32 PFAS standards with ten-calibration points ranging from 0.01 to 20 ppb were used for calibration. A continuing calibration verification standard (CCV) with a known concentration at 5 ppb was injected after every ten samples, along with instrumental blank. Five method blanks (process controls) were included to monitor and reduce the contamination level originated from the instrument itself in potential.

All apparatus and consumables were cleaned and washed previously with MQ water, acetone, and methanol. PP tubes were used, and the TOP assay was conducted under a laminar air-flow cabinet throughout the study. Isotopically labelled internal standard of an appropriate amount (5 ppb) was added to each sample (Supplementary Table S1). An optimal recovery (89%–117%) of each analyte was observed after calculating the recovery of the internal standard by using the mentioned equation in Supplementary Material (Supplementary Equation S1). Additionally, the coefficients correlation (*r* ^*2*^) of the linear calibration curve was observed to be more than ∼0.99.

### 2.6 Data analysis

In this study, a nanomolar (nmol/L or nM) unit was preferred to check the better PFASs recovery analysis rather than μg/L (ppb, parts per billion), as described earlier study by Al Amin et al. (2021). Briefly, nM concentrations of each compound were calculated by dividing the molecular weight of respective compounds after multiplying 1,000× with the concentration (μg/L, or ppb) detected by LC-MS/MS.

$$\text{nM}=1000\times \mu g/L\;\left(\text{ppb}\right)\;/\text{ molecular weight}$$ (1)

Reaction completeness or percentage (%) of oxidation, after TOP assay was assessed according to Slee et al. (2019);

$$\left(\%\right)\text{ of oxidation}=\left(1-\text{sum of precursors }/\text{ sum of PFASs}\right)\times 100$$ (2)

Additionally, the limit of detection (LOD) of all the tested compounds was mentioned in Supplementary Table S2 (Supplementary Material).

Note, although FTS is recommended as an indicator to monitor the oxidation process, its residue amount is accounted for in the sum of PFASs (∑PFASs), along with other oxidation products as well, as suggested below.

## 3 Results and discussion

### 3.1 Initial concentration of AFFFs

The modified TOP assay was applied to oxidise four AFFF samples (#1–4), namely, Orchidee (#1), Wormald (#2), and Ansulite (#3 and #4), which have been used before in Australia. Three sets of dilutions, for instance, 5,00,000×, 20,000×, and 1,000× were chosen, and subjected to the TOP assay to investigate the effect/performance of oxidation on initial concentrations of PFASs in AFFF. The results are presented below and the control tests on PFOS and 6:2 FTS are provided in Supplementary Figure S3 (Supplementary Material).

#### 3.1.1 5,00,000× dilution

**FIGURE 1:**
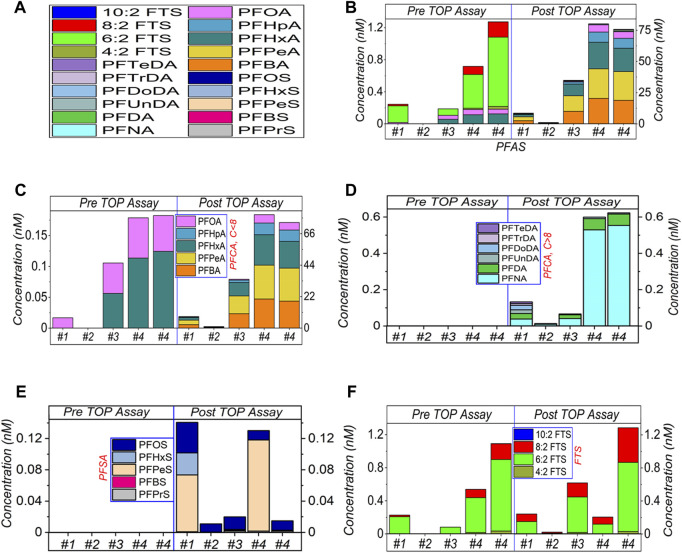
HPLC-MS/MS results of TOP assay of AFFF samples #1 to #4, at 5,00,000× dilution. **(A)** shows the legend of detected PFASs. **(B)** shows the sum amount, and **(C–F)** presents the categorised amounts of the short-chain (≤C8) of PFCA, long-chain (>C8) of PFCA, PFSA and FTS respectively. The empty bars of each figures corresponding the concentrations of the compounds are below the limit of detection.

**FIGURE 2:**
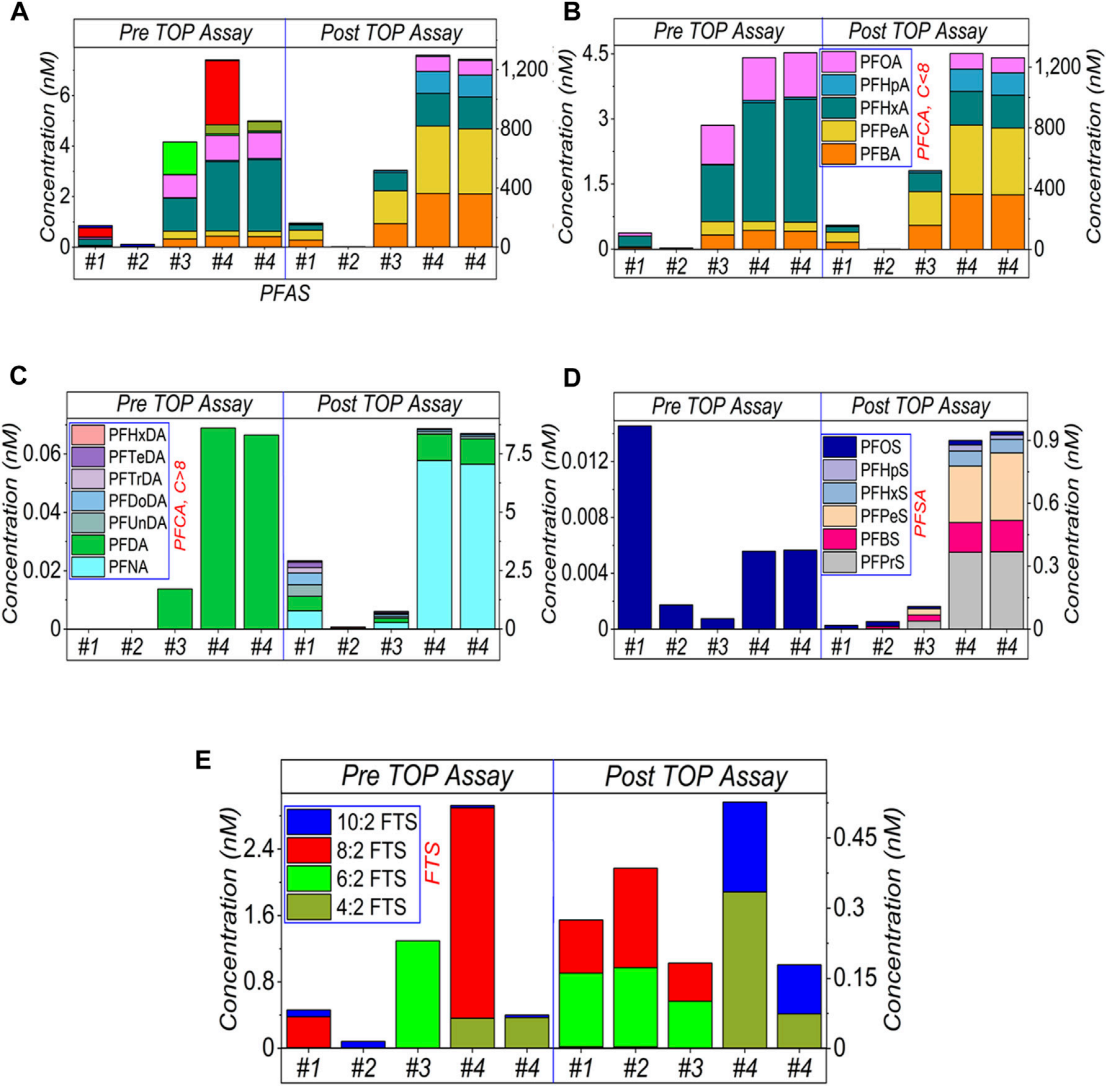
HPLC-MS/MS results of AFFF samples after 20,000× dilution and subject to the TOP assay. **(A)** shows the sum amount while **(B–E)** presents the categorised amounts. Specifically, **(B)** shows the short-chain (≤C8) of PFCA, **(C)** the long-chain (>C8) of PFCA, **(D)** PFSA and **(E)** FTS, respectively. The legends are presented in Figure 1A. The empty bars of each figures corresponding the concentrations of the compounds are below the limit of detection.

**FIGURE 3:**
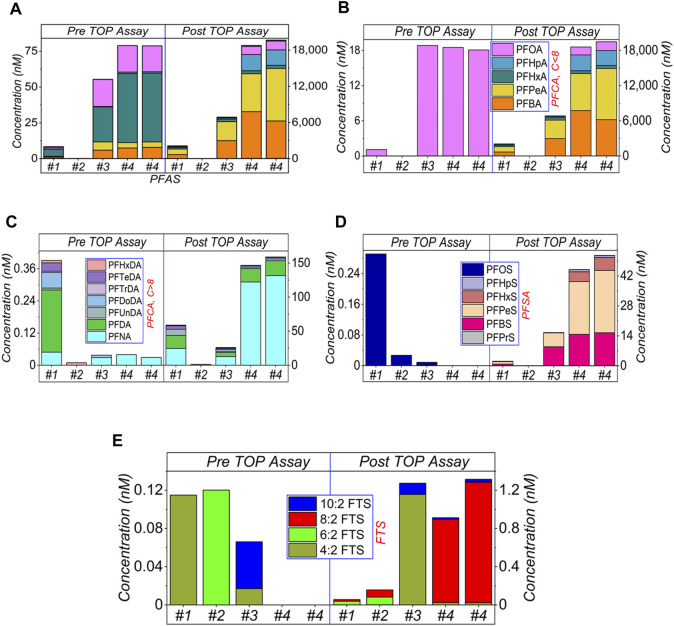
HPLC-MS/MS results of AFFF samples, after 1,000× dilution and subject to the TOP assay. **(A)** shows the sum amount, and **(B–E)** presents the categorised amounts. Specifically, **(B)** shows the short-chain (≤C8) of PFCA, **(C)** the long-chain (>C8) of PFCA, **(D)** PFSA and **(E)** FTS, respectively. The legends are presented in Figure 1A. The empty bars of each figures corresponding the concentrations of the compounds are below the limit of detection.

In Figure 1, four AFFF samples have been diluted by 5,00,000× and subjected to the modified TOP assay. When we targeted and analysed 32 PFASs in this study, 20 PFASs were detected. In Figure 1B, after TOP assay sum of PFASs concentration increased by ∼35 times for #1, ∼534 times for #2, ∼186 times for #3 and 59–112 times for #4 (duplicated, the variation is discussed below), respectively. All data are listed in Supplementary Table S2 (Supplementary Material, mainly used for Figures 1–3).

Figure 1C shows that a significant amount of short-chain PFCA has been released by the TOP assay. Similarly, the long-chain PFCA and the short/long-chain PFSA have also been released, as shown in Figures 1D, E. Obviously, this is due to the oxidation of the PFASs precursors in the AFFF samples. Most of these PFCA and PFSA can be categorised as the TOP assay end products, which means they are stable and cannot be further oxidised by the TOP assay due to the lack of reaction conditions.

In Figure 1F, prior to the TOP assay, FTS molecules were detected in AFFF samples #1, #3 and #4. The highest PFASs concentration in these samples was 6:2 FTS, followed by 8:2 FTS. After the TOP assay, while the concentration of 6:2 FTS decreased in #1 and #4, it increased in #2 and #3. The concentration of 8:2 FTS generally increased after the TOP assay, except #4. Different from PFCA and PFSA, FTS can be further oxidised (Supplementary Figures S1–S3, Supplementary Material). That is, FTS can be further oxidised to decrease the concentration, and can also be released by oxidising the PFASs precursors to increase its concentration. These two processes compete to determine the end concentration of FTS after the TOP assay. In Figure 1F, the increased concentration of 8:2 FTS suggests that its release from the AFFF samples dominates the competing processes. For 6:2 FTS, the released amount looks comparable with the oxidised amount, which leads to the variation in concentration. The different AFFF samples have different backgrounds or complexity, which leads to variations in the FTS concentrations, thus affecting the TOP assay process and oxidation efficiency.

The residual FTS after TOP assay suggests that further oxidation can be conducted, because FTS survived the oxidation, which means other PFASs precursors might also have survived. In this case, the oxidation of the PFASs precursors is potentially incomplete at this dilution factor. In the following sections, we will compare other dilution factors. Overall, the highest amount was noted for PFCA (C_4_–C_15_) (∼200 nM), then followed by FTS (∼2.3 nM) and PFSA (C_5_–C_10_) (∼0.13 nM), at 5,00,000× dilution, which suggests that several intermediates (such as, PFBA, PFPeA, PFHpA etc.) from the non-quantifiable PFAA precursors are yielded during the TOP assay, whereas most of those compounds were not detected in pre-TOP assay (more details in Supplementary Table S2, Supplementary Material).

#### 3.1.2 20,000× dilution

Results for 20,000× dilutions are shown in Figure 2. It was observed that the sum of PFASs after TOP assay in (a), categorised amount of PFCA in (b, c), and PFSA in (d) increased, while different types of FTS (e) decreased. Interestingly, different PFSA (i.e., C3–C7) was yielded in addition to PFOS in (d), perhaps due to the hydrolysis of sulfonamide-based precursors. In Figure 2E, 10:2 FTS has also been detected in AFFF samples #1, #2 and #4 (pre-) and #4 (post TOP assay). A tiny amount of varied FTS residue in the post TOP assay results suggests oxidation occurred or may be impeded due to the dissolved organic matter.

#### 3.1.3 1,000× dilution

Results for 1,000× dilutions are shown in Figure 3. Again, similar phenomena are observed, including the increased amount of PFASs after TOP assay (a), PFCA (b, c), PFSA (d) and varying concentrations of FTS in (e).

For all three sets of dilutions (Figures 1–3), the total PFASs concentrations in all four AFFF samples were increased by the TOP assay, which was dominated by the increased concentrations of PFCAs, particularly short-chain PFCA. However, the ultra-short chain of PFASs was not monitored due to the absence of standards in this study. As mentioned above, PFCA and PFSA were generated as the end products, either from the hydrolysis of sulfonamide precursors of FTS molecules or from other intermediates (Vecitis et al., 2009; Rattanaoudom et al., 2012; Al Amin et al., 2021). However, due to the sample’s complexity, most of the PFASs precursors are likely unidentified at this moment. Hence the reason why TOP assay is required to convert the precursors to detectable PFASs.

As earlier discussed, the conversion and oxidation of the unidentified PFASs precursors may also release FTS as oxidation intermediates (before the release of PFCA) (Gonzalez et al., 2021). The non-C-F moieties in the PFASs precursors can be easily oxidised to release FTS (Harding-Marjanovic et al., 2015; Bruton and Sedlak, 2017; Houtz et al., 2018), and then FTS can be further oxidised to PFCA (Slee et al., 2019). These two processes (to release FTS and to further oxidise FTS) compete and depend on the TOP assay parameters, such as the amount of oxidising reagent, the reaction duration, temperature, and the initial concentration of the analysed samples. This is also why FTS can be recommended as an indicator to monitor the oxidation process. Details are shown in Supplementary Figure S3 (Supplementary Material), where 8:2 FTS was selected as oxidative indicator.

However, there are still some questions on the FTS indicator, including i) oxidation might bypass the intermediate FTS. In this case, FTS can neither be released by PFASs precursors nor act as an indicator; ii) oxidation might not happen at all, such as in Figure 3E, for #2 and #4 before the TOP assay. In this case, the low level of FTS does not mean the oxidation is finished, but the oxidation is not commenced yet; iii) similarly with (ii), a low level of FTS or absence does not indicate the complete oxidation for the TOP assay but suggests the release (by oxidising precursors) is slower than oxidation (of itself). Notably, if the oxidation is not quenched but allowed to continue for a longer time, the continued oxidation can lead to further generation of ultrashort carbon chain PFCA (C < 4) in potential, which currently cannot be effectively and quantitatively detected in this study. Another method is needed to analyse these compounds (Janda et al., 2019).

#### 3.1.4 Comparison of different dilution factors

**FIGURE 4:**
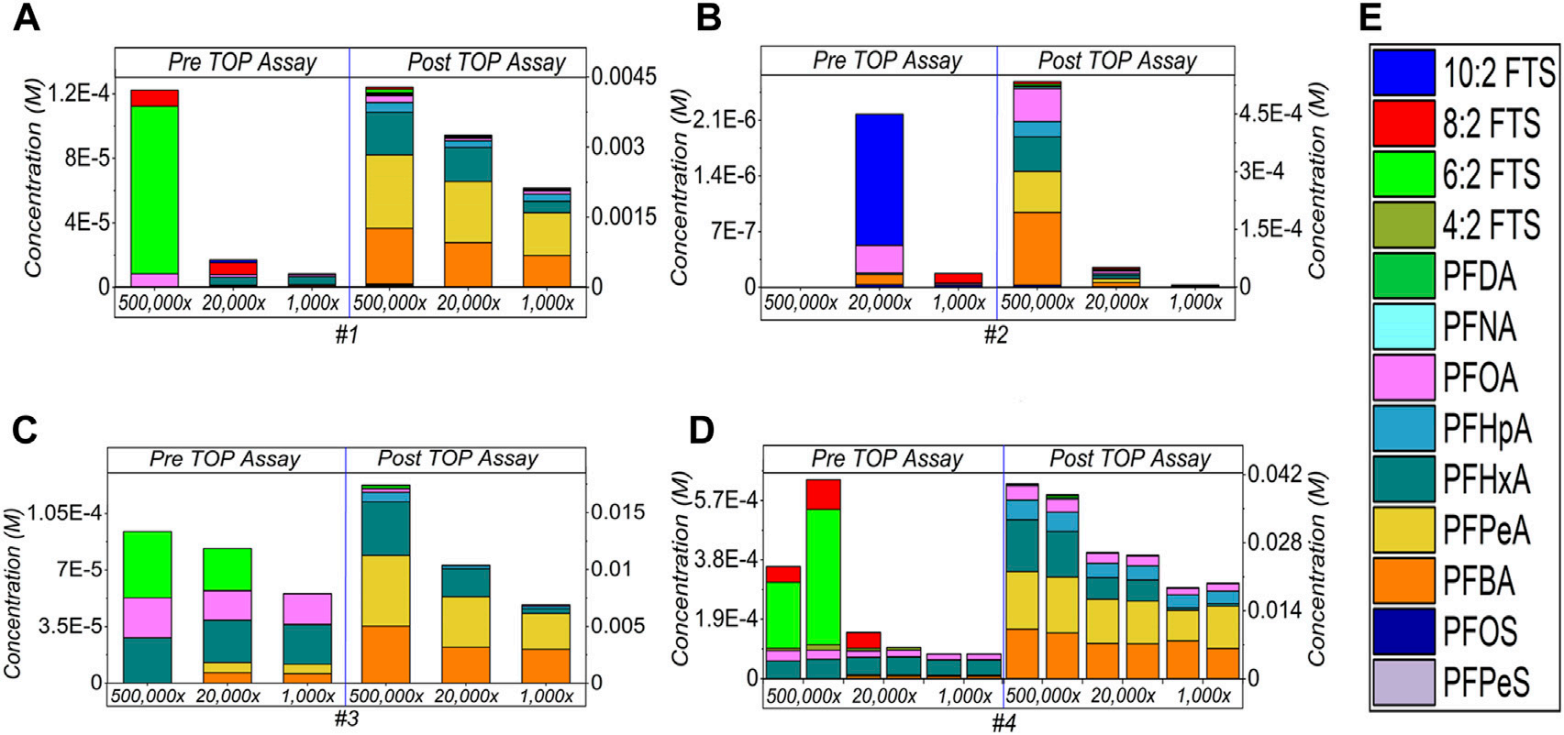
Comparison of the different dilution factors of the AFFF samples [#1–4, for **(A–D)**, respectively] and subject to the TOP assay, and **(E)** demonstrates the detected PFAS compounds by respective colour legend. All concentrations have been converted back to the initial ones by multiplying the dilution factor. The empty bars of each figures corresponding the concentrations of the compounds are below the limit of detection.

After being converted back to the initial concentration by multiplying the dilution factor, Figure 4 shows the comparison study. Different degrees of concentration increase after generating PFASs compounds at 5,00,000×, then followed by 20,000× and 1,000×. Specifically, sample #1 (Figure 4A) shows increase of 35×, 191×, and 252× at 5,00,000×, 20,000×, and 1,000× dilution factors respectively. For sample #2 (Figure 4B), total PFAS concentrations increased to 267×, 23× and 33×, correspondingly. 186×, 125× and 125× increases are counted for sample #3 (Figure 4C), whereas 59–112×, 175–254× and 238–250× are observed for sample #4 and its duplicated one’s (Figure 4D), accordingly. All the data are listed in Supplementary Table S3 (Supplementary Material).

The decline in the sum concentration generated from high to low dilution is expected. That is because at high dilution rate, the concentration of target compounds is low and enables a pronounced attack by the radical towards oxidation. In Figures 1–3 and Figure 4E, it was noticed that the sum of the concentration of PFASs has variations in the duplication of sample #4, ∼47% at 5,00,000×, ∼31% at 20,000× and ∼5% at 1,000×. To generate the reproducible or equivalent conversion of the sum of PFASs in replicated samples during the TOP assay is quite challenging. The reasons could be i) the nature of the TOP assay, that is, the random attack and subsequent oxidation, indicating relatively low reproducibility of results, ii) Other interferences present in the target samples may consume most or all the oxidants, thus interrupting the complete degradation of the target compounds. As a result, proper conversion and appropriate quantification of hidden PFASs molecules are disrupted (Nikiforov, 2021). Therefore, more research is needed here.

### 3.2 TOP assay optimisation

As discussed, FTS should be cautiously selected as the indicator to suggest the oxidation efficiency (Slee et al., 2019). In Figure 1F/2E/3E, we can see the FTS residues for all sets of dilutions. The residual FTS suggests the oxidation can be further improved (Janda et al., 2019; Slee et al., 2019; Gockener et al., 2020; Göckener et al., 2021). In this section, TOP assay is further modified. To mimic the heavily contaminated samples with organic background and to maximise the oxidation capacity of TOP assay, the AFFF samples were diluted to 100× and subjected to TOP assay.

**FIGURE 5:**
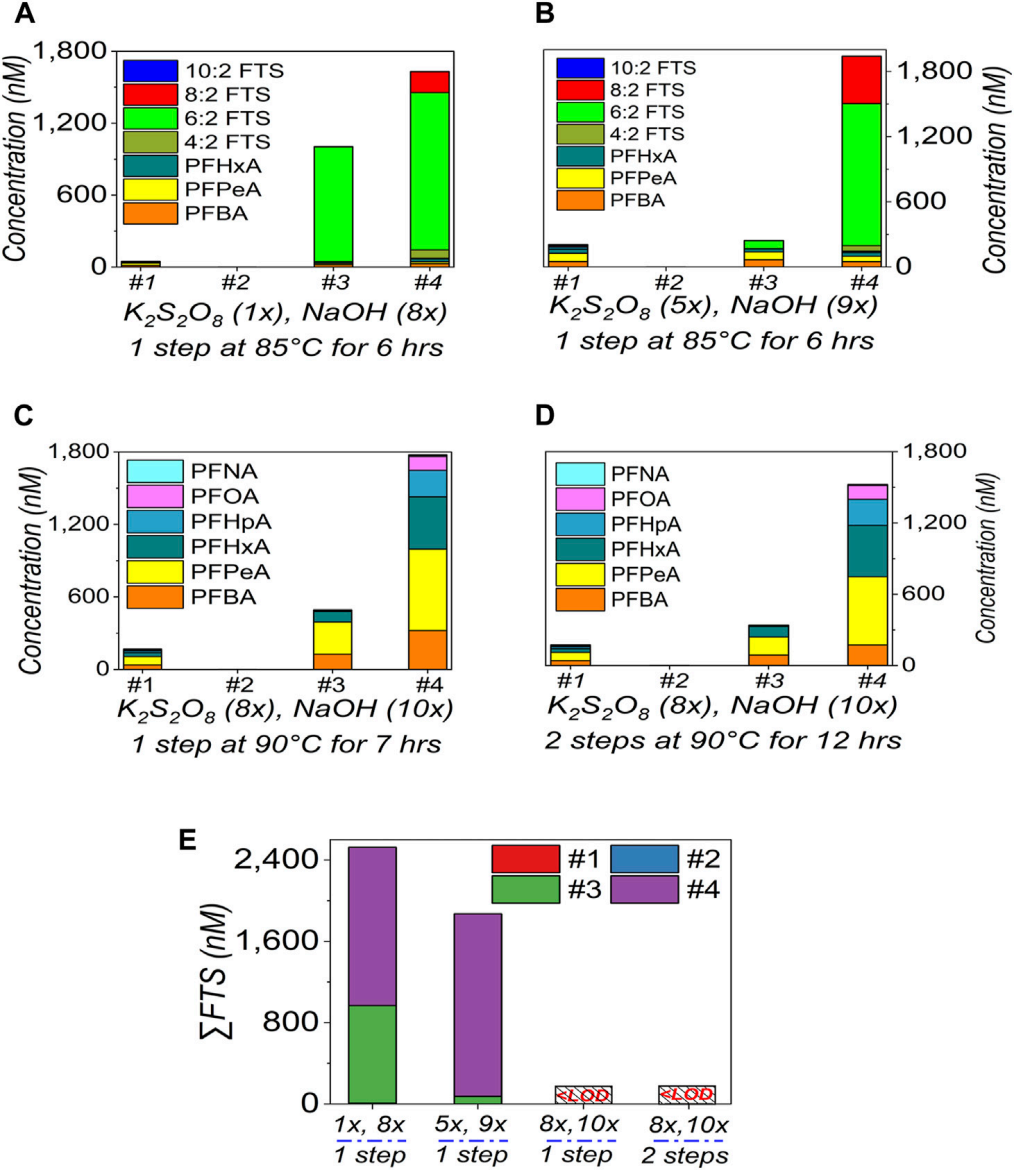
Optimisation on the TOP assay **(A–D)** and residual FTS **(E)** in AFFF. The TOP assay reagents doses are K_2_S_2_O_8_ (1×), NaOH (8×) **(A)**; 5×/9× **(B)**; 8×/10× to conduct the oxidation in 1-step **(C)** or 2-step **(D)** at 90 °C, as marked under each plot. All the AFFF samples were diluted 100× times. Only the main PFASs targets are presented here. In **(E)**, all the data was extracted from **(A–D)**, and “LOD” is the limit of detection (0.01–0.02 ppb). The empty bars of each figures corresponding the concentrations of the compounds are below the limit of detection.

In Figures 5A, B, almost similar results/patterns were observed with the increased doses of K_2_S_2_O_8_ (from 1× to 5×) and NaOH (from 8× to 9×), with 4:2 FTS, 6:2 FTS, and 8:2 FTS dominating the FTS, which means the oxidation is not yet finished, due to the sample complexity at the low dilution factor.

However, in Figures 5C, D, when the extra doses (K_2_S_2_O_8_: from 5× to 8×, NaOH: from 9× to 10×) were introduced at a higher temperature (from 85°C to 90°C), and with a longer oxidation duration (from 6 h to 7 h and to 12 h with 2-step or 2-round of oxidation), FTS has almost disappeared (Supplementary Table S4, Supplementary Material). That is, the TOP assay has been improved to achieve a higher oxidation efficiency, if taking FTS as the indicator. The PFCAs were also effectively released, which generated similar patterns to those in Figures 1–4. We thus select the TOP assay [dose of K_2_S_2_O_8_ (8×), NaOH (10×), 1-step for 7 h] (Figure 5C) for the following test. The 2-step process takes 12 h in Figure 5D so that it is not selected, to shorten the testing duration.

The success of the improved TOP assay on the complex sample (100× dilution here for AFFF samples) leads us to use it for the PFAS-contaminated soil sample in the following section.

### 3.3 PFAS-contaminated soil

**FIGURE 6:**
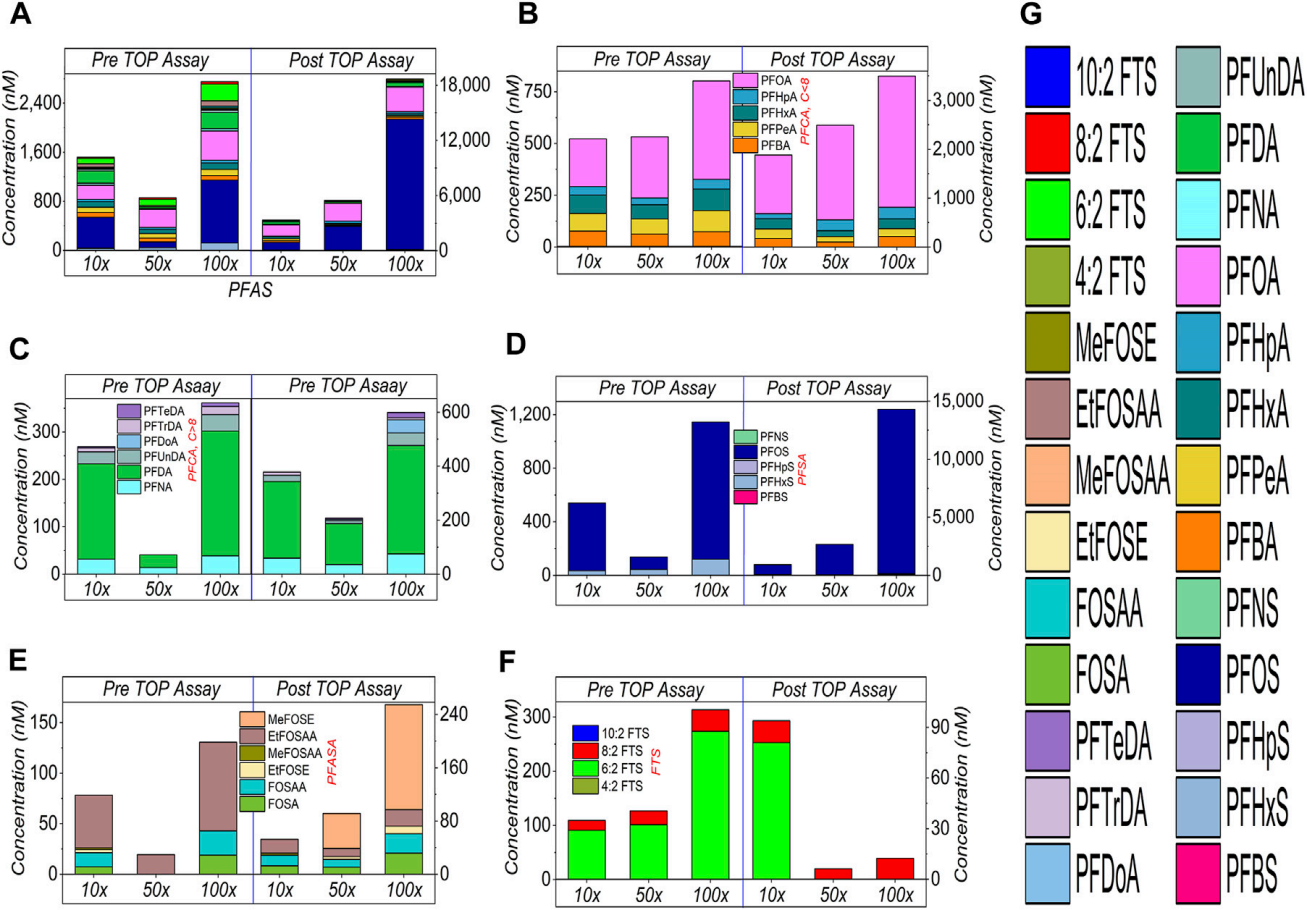
TOP assay analysis on PFASs in the soil at different dilution factors. **(A)** shows the sum of PFASs, and **(B–F)** present the categorised amount. Specifically, **(B)** shows the short-chain (≤C8) PFCA **(B)**, **(C)** lists the long-chain (>C8) PFCA **(C)**, **(D)** PFSA **(D)**, **(E)** PFASAs **(E)**, and **(F)** FTS **(F)**, respectively. Detected PFAS compounds are demonstrated by respective color legend **(G)**.

PFAS and particularly PFAS precursors in the soil cannot be detected effectively, therefore the improved TOP assay was applied to reveal and quantify the hidden PFASs precursors in the PFAS-contaminated soil. The soil was characterised and presented in Supplementary Table S5 (Supplementary Material). In Figure 6G, of the 32 PFASs compounds targeted, 24 PFASs compounds were detected. The sum of the PFASs concentration increased ∼2, ∼6 and ∼7 times by the TOP assay at 10×, 50× and 100× dilution factors (Figure 6A), respectively, which indicates the successful oxidation.

Specifically, in Figure 6, the released PFASs includes PFCA (b, c), PFSA (d), PFASA (e) and FTS (f). In Figure 6F, the FTS (8:2 and 6:2) were detected before the oxidation. For the same reason mentioned above, the oxidation efficiency increases with increasing dilution factor, because all FTS molecules almost completely degraded at 50× and 100× (Figure 6F).

In Figures 6B–D, short-chain and long-chain PFCAs and PFASs have been detected with increased concentrations compared to the pre-TOP assay. While the sorption of PFASs on soil (Zhao et al., 2012) is complicated and beyond the scope of this study (Bolan et al., 2021), the results show the success of the TOP assay on the soil test.

In Figure 6E, perfluorooctane sulfonamides (PFASA), such as *N*-methylperfluorooctane sulfonamidoethanol (MeFOSE), have also been detected in a significant amount. These compounds have been used in the paper-coating industry (Rhoads et al., 2008; Buck et al., 2011), and might lead to soil contamination. These substances survived the TOP assay, which could be released by degrading sulfonamide ethanol-based precursors (Rhoads et al., 2008; Plumlee et al., 2009; Zhao et al., 2016). PFASA survived the TOP assay, most likely experiencing a similar oxidation pathway (competition between being released and being further oxidised) as FTS, shown in Supplementary Figure S3 (Supplementary Material), but needs more research. Fortunately, both PFASA and FTS are detectable and accountable for the sum of PFASs, once subject to the TOP assay. The protocols of the TOP assay to analyse the soil sample is provided in Supplementary Figures S1, S2 (Supplementary Material), and the data are listed in Supplementary Table S6 (Supplementary Material).

According to Eq. 2 (Section 2.6), the oxidation efficiency was estimated at 96%, 98%, and 99% at 10×, 50× and 100× dilution factors, respectively. Comparing these values with the recently inter-laboratory study (Slee et al., 2019), where the TOP assay was also improved with various parameters including oxidant doses, this study provides a comparable or higher oxidation efficiency, suggesting an improved efficiency of the TOP assay. In general, the recommended acceptable oxidation efficiency was in the range of >90%–95%, or the residue of <10%–5% of initial concentration of precursors in post TOP assay, for soil and water test, respectively (HEPA, 2018; Slee et al., 2019). The oxidation efficiency in this study (96%–99%) is within this range, suggesting the success on the soil test.

## 4 Conclusion

The TOP assay was modified and applied to test four AFFF samples and a PFAS-contaminated soil sample. Due to the complexity of the background or the different concentration of target samples, the oxidation efficiency varies. In general, an increased amount of TOP assay reagents and the diluted sample are recommended for the complicated samples, along with the modified protocols.

The TOP assay is not a straightforward method and is a time-consuming process. Consequently, the optimisation is quite challenging, and the oxidation efficiency might depend on i) the initial or stock concentration of the sample, the complex background containing the PFASs and the unidentified precursors; ii) the co-existence of the solvent and organic matters included in the background; iii) ultra-short chain PFAAs (C <4) potentially released from the TOP assay that is not yet tested in this study and needs further research in the future; iv) while the FTS is suggested as the indicator to effectively monitor oxidation process, the trade-off should be considered between the oxidation efficiency (i.e., the more oxidation happens by increasing the reagent dose and temperature, the higher is the oxidation efficiency) and the mass balance (i.e., the more oxidation happens, the lower the overall mass recovery-if the oxidation reaction is not stopped by quenching, the continuing oxidation can release the ultra-short PFAAs in potential that cannot be effectively monitored in this report). Therefore, the TOP assay needs to be modified, and the application depends on the backgrounds of the different matrices. The parameters of the complete oxidation might be varied, based on sample background and complexity which could be constrained for reproducible results. Despite these concerns, the TOP assay is an applicable method to monitor PFASs precursors.

## Data Availability

The original contributions presented in the study are included in the article/Supplementary Material, further inquiries can be directed to the corresponding author.
