# Supplementary material for: A modified TOP assay to detect per- and polyfluoroalkyl substances in aqueous film-forming foams (AFFF) and soil
Source: Front Chem. 2023 Oct 10;11:1141182. doi: 10.3389/fchem.2023.1141182 (PMC10595011; doi:10.3389/fchem.2023.1141182)
Supplement: Supplementary file 1 [file DataSheet1.docx]

A modified TOP assay to detect per- and polyfluoroalkyl substances in aqueous film-forming foams (AFFF) and soil

Md. Al Amin^1, 2^, Yunlong Luo^1, 2^, Feng Shi^1, 2^, Linbo Yu^1, 2^, Yanju Liu^1, 2^, Annette Nolan^3^, Olalekan Simon Awoyemi^1^, Megharaj Mallavarapu^1, 2^, Ravi Naidu^1, 2^, Cheng Fang^1, 2*^

^1^Global Centre for Environmental Remediation (GCER), University of Newcastle, Callaghan NSW 2308, Australia.

^2^Cooperative Research Centre for Contamination Assessment and Remediation of the Environment (CRC CARE), University of Newcastle, Callaghan NSW 2308, Australia.

^3^Ramboll Australia, The Junction NSW 2291, Australia

*Corresponding author: Cheng Fang, Tel: +61 2 4913 8740; Fax: +61 2 4913 8740; E-mail: [cheng.fang@newcastle.edu.au](mailto:cheng.fang@newcastle.edu.au); <https://orcid.org/0000-0002-3526-6613>

***Supporting Information***

Table S1: A list of all analysed PFASs and internal standards in this study

| *Target analytes* | *Acronyms* | *Molecular weight* | *Internal Standards* | *MRM transition*  *(m/z)* | | *Detected sources* | |
| --- | --- | --- | --- | --- | --- | --- | --- |
|  |  |  |  |  |  | *AFFF* | *Soil* |
| **Perfluorocarboxylic acids (PFCAs)** |  | | | *Precursor ion* | *Product ion* |  | |
| Perfluorobutanoic acid | PFBA | 214.04 | ^13^C-PFBA | 213 | 169 | √ | √ |
| Perfluoropentanoic acid | PFPeA | 264.05 | ^13^C-PFPeA | 263 | 219 | √ | √ |
| Perfluorohexanoic acid | PFHxA | 314.05 | ^13^C-PFHxA | 313 | 119 | √ | √ |
| Perfluoroheptanoic acid | PFHpA | 364.06 | ^13^C-PFHpA | 363 | 319 | √ | √ |
| Perfluorooctanoic acid | PFOA | 414.07 | ^13^C-PFOA | 413 | 369 | √ | √ |
| Perfluorononanoic acid | PFNA | 464.08 | ^13^C-PFNA | 463 | 219 | √ | √ |
| Perfluorodecanoic acid | PFDA | 514.08 | ^13^C-PFDA | 513 | 269 | √ | √ |
| Perfluoroundecanoic acid | PFUnDA | 564.09 | ^13^C-PFUnDA | 563 | 269 | √ | √ |
| Perfluorododecanoic acid | PFDoDA | 614.1 | ^13^C-PFDoDA | 613 | 319 | √ | × |
| Perfluorotridecanoic acid | PFTrDA | 664.11 | ^13^C-PFDoDA | 663 | 319 | √ | √ |
| Perfluorotetradecanoic acid | PFTeDA | 714.11 | ^13^C-PFDoDA | 713 | 219 | √ | √ |
| Perfluorohexadecanoic acid | PFHxDA | 814.13 | ^13^C-PFDoDA | 813 | 769 | √ | × |
| **Perfluorosulfonic acids (PFSAs)** |  | | |  |  |  | |
| Perfluoropropane sulfonic acid | PFPrS | 250.09 | ^13^C-PFHxS | 249 | 80 | √ | × |
| Perfluorobutane sulfonic acid | PFBS | 300.1 | ^13^C-PFHxS | 299 | 80 | √ | × |
| Perfluoropentane sulfonic acid | PFPeS | 350.11 | ^13^C-PFHxS | 349 | 80 | √ | √ |
| Perfluorohexane sulfonic acid | PFHxS | 400.12 | ^13^C-PFHxS | 399 | 80 | √ | √ |
| Perfluoroheptane sulfonic acid | PFHpS | 450.12 | ^13^C-PFHxS | 449 | 80 | √ | √ |
| Perfluorooctane sulfonic acid | PFOS | 500.13 | ^13^C-PFOS | 499 |  | √ | √ |
| Perfluorononanesulfonic acid | PFNS | 572.12 | ^13^C-PFOS | 549 | 80 | × | √ |
| Perfluorodecane sulfonic acid | PFDS | 600.15 | ^13^C-PFOS | 599 | 80 | × | × |
| **Fluorotelomer sulfonate (FTS)** |  | | |  |  |  | |
| 4:2 Fluorotelomer sulfonic acid | 4:2 FTS | 328.15 | ^13^C-6:2 FTS | 327 | 81 | √ | × |
| 6:2 Fluorotelomer sulfonic acid | 6:2 FTS | 428.17 | ^13^C-6:2 FTS | 427 | 80 | √ | √ |
| 8:2 Fluorotelomer sulfonic acid | 8:2 FTS | 528.18 | ^13^C-6:2 FTS | 527 | 81 | √ | √ |
| 10:2 Fluorotelomer sulfonic acid | 10:2 FTS | 628.2 | ^13^C-6:2 FTS | 627 | 80 | √ | × |
| **Perfluoroalkyl sulfonamides (PFASAs)** |  | | |  |  |  | |
| Perfluorooctane sulfonamidoacetic acid | FOSAA | 557.18 | ^13^C-FOSAA | 556 | 219 | × | √ |
| N-ethyl perfluorooctane sulfonamidoacetic acid | EtFOSAA | 585.23 | ^13^C-FOSAA | 584 | 419 | × | √ |
| N-methyl perfluorooctane sulfonamidoacetic acid | MeFOSAA | 571.21 | ^13^C-FOSAA | 570 | 219 | × | √ |
| Perfluorooctane sulfonamide | FOSA | 499.15 | ^13^C-FOSA | 498 | 48 | √ | √ |
| N-Methyl perfluorooctane sulfonamide | MeFOSA | 513.17 | ^13^C-FOSA | 512 | 169 | × | × |
| N-Ethyl perfluorooctane sulfonamide | EtFOSA | 527.2 | ^13^C-FOSA | 526 | 169 | × | × |
| N-Methyl perfluorooctane sulfonamidoethanol | MeFOSE | 557.22 | ^13^C-FOSA | 526 | 169 | × | √ |
| N-Ethyl perfluorooctane sulfonamidoethanol | EtFOSE | 571.25 | ^13^C-FOSA | 630 | 59 | × | √ |


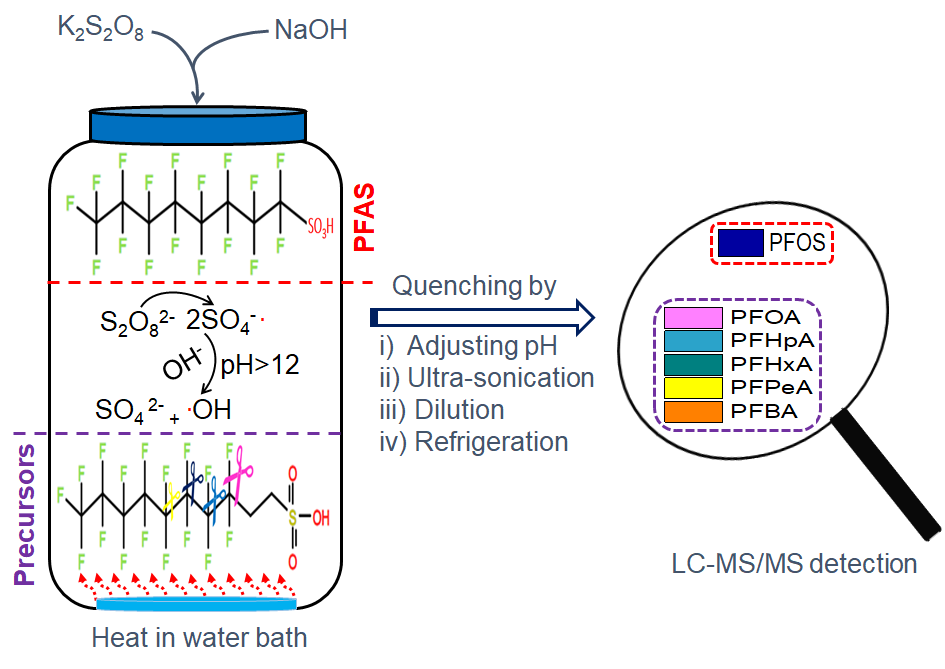
Figure S1: Degradation scenario of C-F and non-C-F moieties of PFAS and PFAS precursors, under the TOP assay at pH >12.

Ideally, the C-F bond (such as in PFAS) can survive the TOP assay. In contrast, the non-C-F moieties (such as deviated in the precursors) are oxidised and randomly attacked by the radicals to release different products.


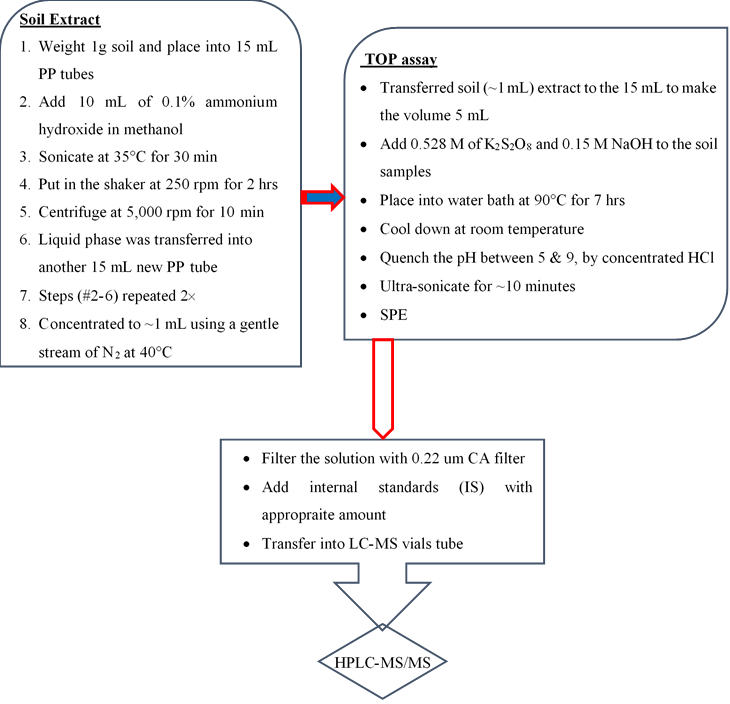


Figure S2: TOP assay protocol for PFAS-contaminated soil test.


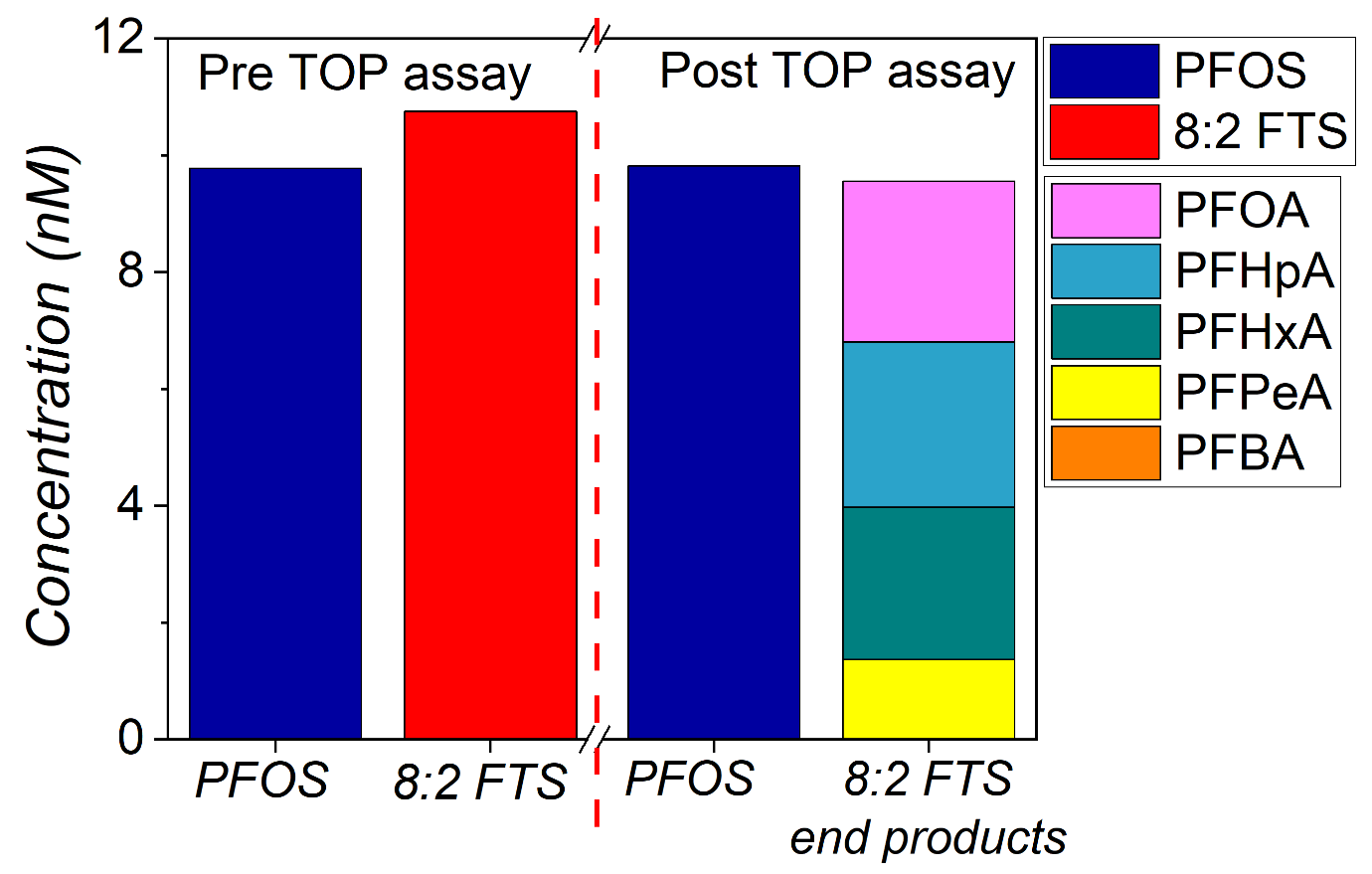


Figure S3: Control analysis of PFOS and 8:2 FTS under the TOP assay.

PFOS was not degraded due to the inert C-F bond/skeleton. However, 8:2 FTS has been oxidised and converted to PFCAs, including pentafluorobenzoic acid **(**PFBA), perfluoropentanoic acid and (PFPeA), perfluoro hexanoic acid (PFHxA), perfluoro heptanoic acid (PFHpA), perfluorooctanoic acid (PFOA), which confirmed the completeness of oxidative reaction. Ultra-short chains of PFCAs (<4) were not detectable here.

Table S2: Concentrations of individual PFAS compounds at three different dilutions of AFFFs samples; data are for Figures 1-3 (the initial samples were diluted and subject to the TOP assay, so that the data below were tabled for the diluted samples, ***NOT*** for the initial / stock samples before the dilution)

|  | ***500,000***× *(For Figure 1)* | | | | | | | | | | | |
| --- | --- | --- | --- | --- | --- | --- | --- | --- | --- | --- | --- | --- |
|  | ***LOD*** | | ***Pre TOP assay [Concentration (nM)]*** | | | | | ***Post TOP assay [Concentration (nM)]*** | | | | |
| ***Detected***  ***compounds*** | ***ppb (μg/L)*** | ***nM*** | ***#1*** | ***#2*** | ***#3*** | ***#4*** | ***#4 (duplicated)*** | ***#1*** | ***#2*** | ***#3*** | ***#4*** | ***#4 (duplicated)*** |
| PFPrS | *0.01* | *0.0400* | *<LOD* | *<LOD* | *<LOD* | *<LOD* | *<LOD* | *<LOD* | *<LOD* | *<LOD* | *<LOD* | *<LOD* |
| PFBS | *0.01* | *0.0334* | *<LOD* | *<LOD* | *<LOD* | *<LOD* | *<LOD* | *<LOD* | *<LOD* | *<LOD* | *<LOD* | *<LOD* |
| PFPeS | *0.01* | *0.0286* | *<LOD* | *<LOD* | *<LOD* | *<LOD* | *<LOD* | 0.0726 | *<LOD* | *<LOD* | *0.117* | *<LOD* |
| PFHxS | *0.01* | *0.0250* | *<LOD* | *<LOD* | *<LOD* | *<LOD* | *<LOD* | 0.0282 | *<LOD* | *<LOD* | *<LOD* | *<LOD* |
| PFHpS | *0.01* | *0.0222* | *<LOD* | *<LOD* | *<LOD* | *<LOD* | *<LOD* | *<LOD* | *<LOD* | *<LOD* | *<LOD* | *<LOD* |
| PFOS | *0.01* | *0.0200* | *<LOD* | *<LOD* | *<LOD* | *<LOD* | *<LOD* | 0.0391 | *<LOD* | *<LOD* | *<LOD* | *<LOD* |
| PFNS | *0.02* | *0.0400* | *<LOD* | *<LOD* | *<LOD* | *<LOD* | *<LOD* | *<LOD* | *<LOD* | *<LOD* | *<LOD* | *<LOD* |
| PFBA | *0.05* | *0.234* | *<LOD* | *<LOD* | *<LOD* | *<LOD* | *<LOD* | 2.39 | 0.378 | 10.0 | 20.3 | 18.9 |
| PFPeA | *0.01* | *0.0379* | *<LOD* | *<LOD* | <LOD | <LOD | <LOD | 3.14 | 0.213 | 12.4 | 23.6 | 23.0 |
| PFHxA | *0.01* | *0.0318* | *<LOD* | *<LOD* | *0.0562* | *0.114* | 0.124 | 1.83 | 0.180 | 9.40 | 21.2 | 18.7 |
| PFHpA | *0.01* | *0.0275* | *<LOD* | *<LOD* | *<LOD* | *<LOD* | *<LOD* | 0.417 | 0.0791 | 1.67 | 8.16 | 7.92 |
| PFOA | *0.01* | *0.0242* | *<LOD* | *<LOD* | *0.0493* | *0.0653* | 0.0580 | 0.286 | 0.172 | 0.623 | 5.84 | 5.28 |
| PFNA | *0.01* | *0.0216* | *<LOD* | *<LOD* | *<LOD* | *<LOD* | *<LOD* | 0.0383 | *<LOD* | 0.0410 | 0.530 | 0.554 |
| PFDA | *0.01* | *0.0195* | *<LOD* | *<LOD* | *<LOD* | *<LOD* | *<LOD* | 0.0301 | *<LOD* | 0.0203 | 0.0614 | 0.0626 |
| PFUnDA | *0.01* | *0.0177* | *<LOD* | *<LOD* | *<LOD* | *<LOD* | *<LOD* | 0.0208 | *<LOD* | *<LOD* | *<LOD* | *<LOD* |
| PFDoDA | *0.01* | *0.0163* | *<LOD* | *<LOD* | *<LOD* | *<LOD* | *<LOD* | 0.0248 | *<LOD* | *<LOD* | *<LOD* | *<LOD* |
| PFTrDA | *0.01* | *0.0151* | *<LOD* | *<LOD* | *<LOD* | *<LOD* | *<LOD* | *<LOD* | *<LOD* | *<LOD* | *<LOD* | *<LOD* |
| PFTeDA | *0.01* | *0.0140* | *<LOD* | *<LOD* | *<LOD* | *<LOD* | *<LOD* | *<LOD* | *<LOD* | *<LOD* | *<LOD* | *<LOD* |
| PFHxDA | *0.01* | *0.0140* | *<LOD* | *<LOD* | *<LOD* | *<LOD* | *<LOD* | *<LOD* | *<LOD* | *<LOD* | *<LOD* | *<LOD* |
| 4:2 FTS | *0.01* | *0.0305* | *<LOD* | *<LOD* | *<LOD* | *<LOD* | 0.0341 | *<LOD* | *<LOD* | *<LOD* | *<LOD* | *<LOD* |
| 6:2 FTS | *0.01* | *0.0234* | 0.208 | *<LOD* | 0.0821 | 0.423 | 0.866 | 0.151 | *<LOD* | 0.430 | 0.116 | 0.838 |
| 8:2 FTS | *0.02* | *0.0380* | *<LOD* | *<LOD* | *<LOD* | 0.101 | 0.190 | 0.0912 | *<LOD* | 0.171 | 0.0858 | 0.419 |
| 10:2 FTS | *0.02* | *0.0318* | *<LOD* | *<LOD* | *<LOD* | *<LOD* | *<LOD* | *<LOD* | *<LOD* | *<LOD* | *<LOD* | *<LOD* |
|  | ***20,000***× *(For Figure 2)* | | | | | | | | | | |  |
|  | ***LOD*** | | ***Pre TOP assay [Concentration (nM)]*** | | | | | ***Post TOP assay [Concentration (nM)]*** | | | | |
| ***Detected***  ***compounds*** | ***ppb (μg/L)*** | ***nM*** | ***#1*** | ***#2*** | ***#3*** | ***#4*** | ***#4 (duplicated)*** | ***#1*** | ***#2*** | ***#3*** | ***#4*** | ***#4 (duplicated)*** |
| PFPrS | *0.01* | *0.0400* | *<LOD* | *<LOD* | *<LOD* | *<LOD* | *<LOD* | *<LOD* | *<LOD* | *<LOD* | 0.366 | 0.3680 |
| PFBS | *0.01* | *0.0334* | *<LOD* | *<LOD* | *<LOD* | *<LOD* | *<LOD* | *<LOD* | *<LOD* | *<LOD* | 0.142 | 0.150 |
| PFPeS | *0.01* | *0.0286* | *<LOD* | *<LOD* | *<LOD* | *<LOD* | *<LOD* | *<LOD* | *<LOD* | *<LOD* | 0.270 | 0.322 |
| PFHxS | *0.01* | *0.0250* | *<LOD* | *<LOD* | *<LOD* | *<LOD* | *<LOD* | *<LOD* | *<LOD* | *<LOD* | 0.0717 | 0.0646 |
| PFHpS | *0.01* | *0.0222* | *<LOD* | *<LOD* | *<LOD* | *<LOD* | *<LOD* | *<LOD* | *<LOD* | *<LOD* | 0.0290 | *<LOD* |
| PFOS | *0.01* | *0.0200* | *LOD* | *LOD* | *LOD* | *LOD* | *LOD* | *LOD* | 0.0223 | *LOD* | 0.0214 | *LOD* |
| PFNS | *0.02* | *0.0400* | *<LOD* | *<LOD* | *<LOD* | *<LOD* | *<LOD* | *<LOD* | *<LOD* | *<LOD* | *<LOD* | *<LOD* |
| PFBA | *0.05* | *0.234* | *<LOD* | *<LOD* | 0.326 | 0.429 | 0.411 | 47.8 | 0.565 | 158 | 362 | 359 |
| PFPeA | *0.01* | *0.0379* | *<LOD* | *<LOD* | 0.308 | 0.208 | 0.211 | 65.4 | 0.493 | 222 | 456 | 440 |
| PFHxA | *0.01* | *0.0318* | 0.248 | *<LOD* | 1.31 | 2.74 | 2.83 | 36.4 | 0.448 | 123 | 222 | 217 |
| PFHpA | *0.01* | *0.0275* | *<LOD* | *<LOD* | *<LOD* | 0.0526 | 0.0521 | 7.19 | 0.224 | 12.9 | 147 | 147 |
| PFOA | *0.01* | *0.0242* | 0.0738 | *<LOD* | 0.902 | 0.984 | 1.02 | 2.66 | 0.319 | 2.31 | 102. | 100 |
| PFNA | *0.01* | *0.0216* | *<LOD* | *<LOD* | *<LOD* | *<LOD* | *<LOD* | 0.774 | *<LOD* | 0.272 | 7.21 | 7.05 |
| PFDA | *0.01* | *0.0195* | *<LOD* | *<LOD* | *<LOD* | 0.0689 | 0.0665 | 0.624 | *<LOD* | 0.189 | 1.14 | 1.08 |
| PFUnDA | *0.01* | *0.0177* | *<LOD* | *<LOD* | *<LOD* | *<LOD* | *<LOD* | 0.495 | *<LOD* | 0.0718 | 0.105 | 0.105 |
| PFDoDA | *0.01* | *0.0163* | *<LOD* | *<LOD* | *<LOD* | *<LOD* | *<LOD* | 0.507 | *<LOD* | 0.0945 | 0.0828 | 0.0844 |
| PFTrDA | *0.01* | *0.0151* | *<LOD* | *<LOD* | *<LOD* | *<LOD* | *<LOD* | 0.226 | *<LOD* | 0.0418 | 0.0172 | 0.0170 |
| PFTeDA | *0.01* | *0.0140* | *<LOD* | *<LOD* | *<LOD* | *<LOD* | *<LOD* | 0.228 | *<LOD* | 0.0546 | 0.0240 | 0.0248 |
| PFHxDA | *0.01* | *0.0140* | *<LOD* | *<LOD* | *<LOD* | *<LOD* | *<LOD* | 0.0525 | 0.0525 | 0.0209 | *<LOD* | *<LOD* |
| 4:2 FTS | *0.01* | *0.0305* | *<LOD* | *<LOD* | *<LOD* | 0.361 | 0.370 | *<LOD* | *<LOD* | *<LOD* | 0.335 | *<LOD* |
| 6:2 FTS | *0.01* | *0.0234* | *<LOD* | *<LOD* | 1.29 | *<LOD* | *<LOD* | 0.158 | 0.170 | 0.100 | *<LOD* | *<LOD* |
| 8:2 FTS | *0.02* | *0.0380* | 0.372 | *<LOD* | *<LOD* | 2.54 | *<LOD* | 0.114 | 0.212 | 0.0814 | *<LOD* | *<LOD* |
| 10:2 FTS | *0.02* | *0.0318* | 0.0824 | 0.0824 | *<LOD* | *<LOD* | *<LOD* | *<LOD* | *<LOD* | *<LOD* | 0.192 | 0.105 |
|  | ***1,000***× *(For Figure 3)* | | | | | | | | | | |  |
|  | ***LOD*** | | ***Pre TOP assay [Concentration (nM)]*** | | | | | ***Post TOP assay [Concentration (nM)]*** | | | | |
| ***Detected***  ***compounds*** | ***ppb (μg/L)*** | ***nM*** | ***#1*** | ***#2*** | ***#3*** | ***#4*** | ***#4 (duplicated)*** | ***#1*** | ***#2*** | ***#3*** | ***#4*** | ***#4 (duplicated)*** |
| PFPrS | *0.01* | *0.0400* | *<LOD* | *<LOD* | *<LOD* | *<LOD* | *<LOD* | *<LOD* | *<LOD* | *<LOD* | *<LOD* | *<LOD* |
| PFBS | *0.01* | *0.0334* | *<LOD* | *<LOD* | *<LOD* | *<LOD* | *<LOD* | 0.768 | *<LOD* | 8.92 | 14.7 | 15.4 |
| PFPeS | *0.01* | *0.0286* | *<LOD* | *<LOD* | *<LOD* | *<LOD* | *<LOD* | 1.18 | *<LOD* | 6.43 | 24.6 | 29.1 |
| PFHxS | *0.01* | *0.0250* | *<LOD* | *<LOD* | *<LOD* | *<LOD* | *<LOD* | 0.134 | *<LOD* | *<LOD* | 4.55 | 6.08 |
| PFHpS | *0.01* | *0.0222* | *<LOD* | *<LOD* | *<LOD* | *<LOD* | *<LOD* | 0.0317 | *<LOD* | 0.0616 | 0.957 | 0.820 |
| PFOS | *0.01* | *0.0200* | 0.291 | *<LOD* | *<LOD* | *<LOD* | *<LOD* | 0.0280 | 0.0344 | 0.0938 | 0.111 | 0.104 |
| PFNS | *0.02* | *0.0400* | *<LOD* | *<LOD* | *<LOD* | *<LOD* | *<LOD* | *<LOD* | *<LOD* | *<LOD* | *<LOD* | *<LOD* |
| PFBA | *0.05* | *0.234* | 0.878 | *<LOD* | 5.92 | 7.44 | 7.89 | 678 | 1.27 | 2970 | 7760 | 6200 |
| PFPeA | *0.01* | *0.0379* | 0.426 | *<LOD* | 5.78 | 3.76 | 3.76 | 916 | 1.38 | 3140 | 6290 | 8720 |
| PFHxA | *0.01* | *0.0318* | 5.05 | *<LOD* | 24.4 | 48.2 | 48.0 | 245 | 0.914 | 429 | 497 | 508 |
| PFHpA | *0.01* | *0.0275* | 0.159 | *<LOD* | 0.264 | 0.977 | 0.988 | 152 | 0.396 | 189 | 2680 | 2570 |
| PFOA | *0.01* | *0.0242* | 1.10 | *<LOD* | 18.8 | 18.5 | 18.0 | 69.1 | 0.455 | 118 | 1360 | 1480 |
| PFNA | *0.01* | *0.0216* | 0.0486 | *<LOD* | 0.0283 | 0.0395 | 0.0291 | 24.5 | 0.0268 | 12.5 | 122 | 132 |
| PFDA | *0.01* | *0.0195* | 0.232 | *<LOD* | *<LOD* | *<LOD* | *<LOD* | 19.3 | *<LOD* | 6.11 | 19.6 | 21.7 |
| PFUnDA | *0.01* | *0.0177* | *<LOD* | *<LOD* | *<LOD* | *<LOD* | *<LOD* | 8.96 | *<LOD* | 1.93 | 2.27 | 2.68 |
| PFDoDA | *0.01* | *0.0163* | 0.0579 | *<LOD* | *<LOD* | *<LOD* | *<LOD* | *<LOD* | *<LOD* | 2.72 | 1.79 | 1.96 |
| PFTrDA | *0.01* | *0.0151* | *<LOD* | *<LOD* | *<LOD* | *<LOD* | *<LOD* | *<LOD* | *<LOD* | 1.05 | 0.337 | 0.366 |
| PFTeDA | *0.01* | *0.0140* | 0.0310 | *<LOD* | *<LOD* | *<LOD* | *<LOD* | 5.12 | *<LOD* | 1.17 | 0.391 | 0.431 |
| PFHxDA | *0.01* | *0.0140* | *<LOD* | *<LOD* | *<LOD* | *<LOD* | *<LOD* | 0.918 | 0.918 | 0.388 | 0.0630 | 0.0706 |
| 4:2 FTS | *0.01* | *0.0305* | 0.115 | *<LOD* | *<LOD* | *<LOD* | *<LOD* | *<LOD* | *<LOD* | 1.16 | *<LOD* | *<LOD* |
| 6:2 FTS | *0.01* | *0.0234* | *<LOD* | 0.120 | *<LOD* | *<LOD* | *<LOD* | 0.0380 | 0.0800 | *<LOD* | *<LOD* | *<LOD* |
| 8:2 FTS | *0.02* | *0.0380* | *<LOD* | *<LOD* | *<LOD* | *<LOD* | *<LOD* | *<LOD* | 0.0759 | *<LOD* | 0.872 | 1.26 |
| 10:2 FTS | *0.02* | *0.0318* | *<LOD* | *<LOD* | 0.0492 | *<LOD* | *<LOD* | *<LOD* | *<LOD* | 0.118 | *<LOD* | *<LOD* |

***Note:*** *LOD, Limit of Detection; ppb, parts per billion, µg/L; nM, NanoM, nanomol/L*

Table S3: Concentrations of individual PFAS compounds at three different dilutions of AFFFs samples; data are for Figure 4 (all the data below were tabled for the stock samples, after being converted back to the initial ones by multiplying the respective dilution factors)

|  | ***#1*** *(For Figure 4a)* | | | | | | | |
| --- | --- | --- | --- | --- | --- | --- | --- | --- |
|  | ***LOD*** | | ***Pre TOP assay*** ***[Concentration (M)]*** | | | ***Post TOP assay [Concentration (M)]*** | | |
| ***Detected***  ***compounds*** | ***ppb (μg/L)*** | ***M (mol/L)*** | ***500,000×*** | ***20,000×*** | ***1,000×*** | ***500,000×*** | ***20,000×*** | ***1,000×*** |
| PFBS | *0.01* | *3.34E-11* | *<LOD* | *<LOD* | *<LOD* | 3.91E-07 | *<LOD* | 7.68E-07 |
| PFPeS | *0.01* | *2.86E-11* | *<LOD* | *<LOD* | *<LOD* | 3.63E-05 | 2.06E-08 | 1.18E-06 |
| PFHxS | *0.01* | *2.50E-11* | *<LOD* | *<LOD* | *<LOD* | 1.41E-05 | *<LOD* | 1.34E-07 |
| PFHpS | *0.01* | *2.22E-11* | *<LOD* | *<LOD* | *<LOD* | *<LOD* | *<LOD* | 3.17E-08 |
| PFOS | *0.01* | *2.00E-11* | *<LOD* | 2.91E-07 | 2.91E-07 | 1.95E-05 | 3.29E-07 | 2.79E-08 |
| PFBA | *0.01* | *2.34E-10* | *<LOD* | 6.28E-07 | 8.78E-07 | 1.19E-03 | 9.56E-04 | 6.78E-04 |
| PFPeA | *0.05* | *3.79E-11* | *<LOD* | 4.45E-07 | 4.26E-07 | 1.57E-03 | 1.31E-03 | 9.16E-04 |
| PFHxA | *0.01* | *3.18E-11* | *<LOD* | 4.95E-06 | 5.05E-06 | 9.13E-04 | 7.30E-04 | 2.45E-04 |
| PFHpA | *0.01* | *2.75E-11* | *<LOD* | *<LOD* | 1.59E-07 | 2.08E-04 | 1.44E-04 | 1.53E-04 |
| PFOA | *0.01* | *2.42E-11* | 8.39E-06 | 1.48E-06 | 1.11E-06 | 1.43E-04 | 5.32E-05 | 6.91E-05 |
| PFNA | *0.01* | *2.16E-11* | *<LOD* | *<LOD* | 4.86E-08 | 1.91E-05 | 1.55E-05 | 2.45E-05 |
| PFDA | *0.01* | *1.95E-11* | *<LOD* | *<LOD* | 2.32E-07 | 1.51E-05 | 1.25E-05 | 1.92E-05 |
| PFUnDA | *0.01* | *1.77E-11* | *<LOD* | *<LOD* | 7.21E-09 | 1.04E-05 | 9.89E-06 | 8.96E-06 |
| PFDoDA | *0.01* | *1.63E-11* | *<LOD* | *<LOD* | 5.79E-08 | 1.24E-05 | 1.01E-05 | *<LOD* |
| PFTrDA | *0.01* | *1.51E-11* | *<LOD* | *<LOD* | 4.89E-09 | 4.74E-06 | 4.51E-06 | *<LOD* |
| PFTeDA | *0.01* | *1.40E-11* | *<LOD* | *<LOD* | 3.10E-08 | 4.66E-06 | 4.56E-06 | 5.12E-06 |
| PFHxDA | *0.01* | *1.40E-11* | *<LOD* | *<LOD* | 9.25E-09 | *<LOD* | 1.05E-06 | 9.18E-07 |
| 4:2 FTS | *0.01* | *3.05E-11* | *<LOD* | 1.32E-07 | 1.15E-07 | *<LOD* | 7.02E-08 | 2.15E-10 |
| 6:2 FTS | *0.01* | *2.34E-11* | 1.04E-04 | *<LOD* | *<LOD* | 7.55E-05 | 3.15E-06 | 3.80E-08 |
| 8:2 FTS | *0.01* | *3.80E-11* | 9.91E-06 | 7.44E-06 | *<LOD* | 4.56E-05 | 2.27E-06 | 1.78E-08 |
| 10:2 FTS | *0.02* | *3.18E-11* | *<LOD* | 1.65E-06 | *<LOD* | *<LOD* | *<LOD* | *<LOD* |

|  | ***#2*** *(For Figure 4b)* | | | | | | | |
| --- | --- | --- | --- | --- | --- | --- | --- | --- |
|  | ***LOD*** | | ***Pre TOP assay [Concentration (M)]*** | | | ***Post TOP assay [Concentration (M)]*** | | |
| ***Detected***  ***compounds*** | ***ppb (μg/L)*** | ***M (mol/L)*** | ***500,000×*** | ***20,000×*** | ***1,000×*** | ***500,000×*** | ***20,000×*** | ***1,000×*** |
| PFPrS | *0.01* | *4.00E-11* | *<LOD* | *<LOD* | *<LOD* | *<LOD* | 3.40E-08 | 2.57E-09 |
| PFBS | *0.01* | *3.34E-11* | *<LOD* | *<LOD* | *<LOD* | *<LOD* | 2.04E-07 | 4.67E-09 |
| PFPeS | *0.01* | *2.86E-11* | *<LOD* | *<LOD* | *<LOD* | *<LOD* | 2.44E-08 | 2.35E-09 |
| PFHxS | *0.01* | *2.50E-11* | *<LOD* | *<LOD* | *<LOD* | *<LOD* | *<LOD* | 1.37E-08 |
| PFHpS | *0.01* | *2.22E-11* | *<LOD* | *<LOD* | *<LOD* | *<LOD* | *<LOD* | *<LOD* |
| PFOS | *0.01* | *2.00E-11* | *<LOD* | 3.48E-08 | 2.71E-08 | *<LOD* | 4.45E-07 | 3.44E-08 |
| PFNS | *0.02* | *4.00E-11* | *<LOD* | *<LOD* | *<LOD* | *<LOD* | *<LOD* | 1.39E-09 |
| PFBA | *0.05* | *2.34E-10* | *<LOD* | 1.27E-07 | *<LOD* | 1.89E-04 | 1.13E-05 | 1.27E-06 |
| PFPeA | *0.01* | *3.79E-11* | *<LOD* | *<LOD* | *<LOD* | 1.07E-04 | 9.85E-06 | 1.37E-06 |
| PFHxA | *0.01* | *3.18E-11* | *<LOD* | *<LOD* | *<LOD* | 9.01E-05 | 8.96E-06 | 9.14E-07 |
| PFHpA | *0.01* | *2.75E-11* | *<LOD* | 1.81E-08 | *<LOD* | 3.95E-05 | 4.48E-06 | 3.96E-07 |
| PFOA | *0.01* | *2.42E-11* | *<LOD* | 3.48E-07 | 1.95E-08 | 8.59E-05 | 6.38E-06 | 4.55E-07 |
| PFNA | *0.01* | *2.16E-11* | *<LOD* | *<LOD* | *<LOD* | 4.46E-06 | 2.96E-07 | 2.68E-08 |
| PFDA | *0.01* | *1.95E-11* | *<LOD* | *<LOD* | *<LOD* | 2.20E-06 | 1.58E-07 | 9.68E-09 |
| PFUnDA | *0.01* | *1.77E-11* | *<LOD* | *<LOD* | *<LOD* | *<LOD* | *<LOD* | *<LOD* |
| PFDoDA | *0.01* | *1.63E-11* | *<LOD* | *<LOD* | *<LOD* | *<LOD* | *<LOD* | 3.94E-09 |
| PFTrDA | *0.01* | *1.51E-11* | *<LOD* | *<LOD* | *<LOD* | *<LOD* | *<LOD* | *<LOD* |
| PFTeDA | *0.01* | *1.40E-11* | *<LOD* | *<LOD* | *<LOD* | *<LOD* | *<LOD* | *<LOD* |
| PFHxDA | *0.01* | *1.40E-11* | *<LOD* | *<LOD* | 9.25E-09 | *<LOD* | 1.05E-06 | 9.18E-07 |
| 4:2 FTS | *0.01* | *3.05E-11* | *<LOD* | *<LOD* | *<LOD* | *<LOD* | 6.79E-08 | 1.29E-09 |
| 6:2 FTS | *0.01* | *2.34E-11* | *<LOD* | *<LOD* | *<LOD* | 5.26E-06 | 3.39E-06 | 8.00E-08 |
| 8:2 FTS | *0.02* | *3.80E-11* | *<LOD* | *<LOD* | 1.20E-07 | 6.25E-06 | 4.25E-06 | 7.59E-08 |
| 10:2 FTS | *0.02* | *3.18E-11* | *<LOD* | 1.65E-06 | *<LOD* | *<LOD* | *<LOD* | *<LOD* |

|  | ***#3*** *(For Figure 4c)* | | | | | | | |
| --- | --- | --- | --- | --- | --- | --- | --- | --- |
|  | ***LOD*** | | ***Pre TOP assay [Concentration (M)]*** | | | ***Post TOP assay [Concentration (M)]*** | | |
| ***Detected***  ***compounds*** | ***ppb (μg/L)*** | ***M (mol/L)*** | ***500,000×*** | ***20,000×*** | ***1,000×*** | ***500,000×*** | ***20,000×*** | ***1,000×*** |
| PFPrS | *0.01* | *4.00E-11* | *<LOD* | *<LOD* | 1.60E-09 | 8.91E-07 | 7.53E-07 | *<LOD* |
| PFBS | *0.01* | *3.34E-11* | *<LOD* | *<LOD* | *<LOD* | 4.44E-07 | 5.94E-07 | 8.92E-06 |
| PFPeS | *0.01* | *2.86E-11* | *<LOD* | *<LOD* | *<LOD* | 5.42E-07 | 5.95E-07 | 6.43E-06 |
| PFHpS | *0.01* | *2.50E-11* | *<LOD* | *<LOD* | *<LOD* | *<LOD* | *<LOD* | 6.16E-08 |
| PFOS | *0.01* | *2.00E-11* | *<LOD* | 1.50E-08 | 7.32E-09 | 8.07E-06 | 2.14E-07 | 9.38E-08 |
| PFNS | *0.02* | *4.00E-11* | *<LOD* | *<LOD* | *<LOD* | *<LOD* | *<LOD* | 1.32E-08 |
| PFBA | *0.05* | *2.34E-10* | *<LOD* | 6.52E-06 | 5.92E-06 | 5.02E-03 | 3.18E-03 | 2.97E-03 |
| PFPeA | *0.01* | *3.79E-11* | *<LOD* | 6.16E-06 | 5.78E-06 | 6.22E-03 | 4.43E-03 | 3.14E-03 |
| PFHxA | *0.01* | *3.18E-11* | 2.81E-05 | 2.61E-05 | 2.44E-05 | 4.70E-03 | 2.47E-03 | 4.30E-04 |
| PFHpA | *0.01* | *2.75E-11* | *<LOD* | 2.87E-07 | 2.64E-07 | 8.37E-04 | 2.59E-04 | 1.89E-04 |
| PFOA | *0.01* | *2.42E-11* | 2.47E-05 | 1.80E-05 | 1.88E-05 | 3.11E-04 | 4.63E-05 | 1.19E-04 |
| PFNA | *0.01* | *2.16E-11* | *<LOD* | *<LOD* | 2.83E-08 | 2.05E-05 | 5.44E-06 | 1.25E-05 |
| PFDA | *0.01* | *1.95E-11* | *<LOD* | 2.75E-07 | *<LOD* | 1.02E-05 | 3.77E-06 | 6.11E-06 |
| PFUnDA | *0.01* | *1.77E-11* | *<LOD* | *<LOD* | *<LOD* | 2.43E-06 | 1.44E-06 | 1.92E-06 |
| PFDoDA | *0.01* | *1.63E-11* | *<LOD* | *<LOD* | 9.13E-09 | *<LOD* | 1.89E-06 | 2.72E-06 |
| PFTrDA | *0.01* | *1.51E-11* | *<LOD* | *<LOD* | *<LOD* | *<LOD* | 8.36E-07 | 1.05E-06 |
| PFTeDA | *0.01* | *1.40E-11* | *<LOD* | *<LOD* | *<LOD* | *<LOD* | 1.09E-06 | 1.16E-06 |
| PFHxDA | *0.01* | *1.40E-11* | *<LOD* | *<LOD* | *<LOD* | *<LOD* | 4.18E-07 | 3.88E-07 |
| 4:2 FTS | *0.01* | *3.05E-11* | *<LOD* | *<LOD* | 1.70E-08 | 9.02E-06 | 1.57E-08 | 1.16E-06 |
| 6:2 FTS | *0.01* | *2.34E-11* | 4.11E-05 | 2.59E-05 | *<LOD* | 2.15E-04 | 2.00E-06 | *<LOD* |
| 8:2 FTS | *0.02* | *3.80E-11* | *<LOD* | *<LOD* | *<LOD* | 8.52E-05 | 1.63E-06 | *<LOD* |
| 10:2 FTS | *0.02* | *3.18E-11* | *<LOD* | *<LOD* | 4.91E-08 | *<LOD* | *<LOD* | 1.18E-07 |
| FOSA | *0.01* | *2.00E-11* | *<LOD* | *<LOD* | *<LOD* | 4.51E-07 | *<LOD* | *<LOD* |

|  | ***#4*** *(For Figure 4d) (with duplicated tests)* | | | | | | | | | | | | | |
| --- | --- | --- | --- | --- | --- | --- | --- | --- | --- | --- | --- | --- | --- | --- |
|  | ***LOD*** | | ***Pre TOP assay [Concentration (M)]*** | | | | | | ***Post TOP assay [Concentration (M)]*** | | | | | |
| ***Detected***  ***compounds*** | ***ppb (μg/L)*** | ***M (mol/L)*** | ***500,000***× | ***500,000***× | ***20,000***× | ***20,000***× | ***1,000***× | ***1,000***× | ***500,000***× | ***500,000***× | ***20,000***× | ***20,000***× | ***1,000***× | ***1,000***× |
| PFPrS | *0.01* | *4.00E-11* | *<LOD* | *<LOD* | *<LOD* | *<LOD* | *<LOD* | *<LOD* | 8.16E-07 | 8.45E-07 | 7.32E-06 | 7.36E-06 | *<LOD* | *<LOD* |
| PFBS | *0.01* | *3.34E-11* | *<LOD* | *<LOD* | *<LOD* | *<LOD* | *<LOD* | *<LOD* | *<LOD* | 3.37E-08 | 2.84E-06 | 3.01E-06 | 1.47E-05 | 1.55E-05 |
| PFPeS | *0.01* | *2.86E-11* | *<LOD* | *<LOD* | *<LOD* | *<LOD* | *<LOD* | *<LOD* | 5.82E-05 | 5.13E-07 | 5.40E-06 | 6.44E-06 | 2.47E-05 | 2.91E-05 |
| PFHxS | *0.01* | *2.50E-11* | *<LOD* | *<LOD* | *<LOD* | *<LOD* | *<LOD* | *<LOD* | *<LOD* | *<LOD* | 1.43E-06 | 1.30E-06 | 4.55E-06 | 6.10E-06 |
| PFHpS | *0.01* | *2.22E-11* | *<LOD* | *<LOD* | *<LOD* | *<LOD* | *<LOD* | *<LOD* | *<LOD* | *<LOD* | 5.81E-07 | 4.26E-07 | 9.57E-07 | 8.21E-07 |
| PFOS | *0.01* | *2.00E-11* | *<LOD* | *<LOD* | 1.11E-07 | 1.13E-07 | *<LOD* | *<LOD* | 5.96E-06 | 6.06E-06 | 4.29E-07 | 3.20E-07 | 1.11E-07 | 1.04E-07 |
| PFNS | *0.02* | *4.00E-11* | *<LOD* | *<LOD* | *<LOD* | *<LOD* | *<LOD* | *<LOD* | *<LOD* | *<LOD* | *<LOD* | *<LOD* | 1.49E-08 | 1.25E-08 |
| PFBA | *0.05* | *2.34E-10* | *<LOD* | *<LOD* | 8.58E-06 | 8.22E-06 | 7.44E-06 | 7.89E-06 | 1.01E-02 | 9.42E-03 | 7.25E-03 | 7.18E-03 | 7.76E-03 | 6.20E-03 |
| PFPeA | *0.01* | *3.79E-11* | *<LOD* | *<LOD* | 4.16E-06 | 4.23E-06 | 3.76E-06 | 3.75E-06 | 1.18E-02 | 1.15E-02 | 9.12E-03 | 8.80E-03 | 6.23E-03 | 8.72E-03 |
| PFHxA | *0.01* | *3.18E-11* | 5.68E-05 | 6.22E-05 | 5.48E-05 | 5.66E-05 | 4.82E-05 | 4.80E-05 | 1.06E-02 | 9.36E-03 | 4.43E-03 | 4.33E-03 | 4.97E-04 | 5.08E-04 |
| PFHpA | *0.01* | *2.75E-11* | *<LOD* | *<LOD* | 1.05E-06 | 1.04E-06 | 9.77E-07 | 9.88E-07 | 4.08E-03 | 3.96E-03 | 2.94E-03 | 2.95E-03 | 2.70E-03 | 2.60E-03 |
| PFOA | *0.01* | *2.42E-11* | 3.26E-05 | 2.9E-05 | 1.97E-05 | 2.04E-05 | 1.85E-05 | 1.81E-05 | 2.92E-03 | 2.64E-03 | 2.04E-03 | 1.98E-03 | 1.36E-03 | 1.48E-03 |
| PFNA | *0.01* | *2.16E-11* | *<LOD* | *<LOD* | *<LOD* | *<LOD* | 3.95E-08 | 2.91E-08 | 2.65E-04 | 2.77E-04 | 1.44E-04 | 1.41E-04 | 1.22E-04 | 1.31E-04 |
| PFDA | *0.01* | *1.95E-11* | *<LOD* | *<LOD* | 1.38E-06 | 1.33E-06 | *<LOD* | *<LOD* | 3.07E-05 | 3.13E-05 | 2.27E-05 | 2.17E-05 | 1.96E-05 | 2.17E-05 |
| PFUnDA | *0.01* | *1.77E-11* | *<LOD* | *<LOD* | *<LOD* | *<LOD* | *<LOD* | *<LOD* | 4.22E-06 | 3.40E-06 | 2.10E-06 | 2.10E-06 | 2.27E-06 | 2.69E-06 |
| PFDoDA | *0.01* | *1.63E-11* | *<LOD* | *<LOD* | *<LOD* | *<LOD* | *<LOD* | *<LOD* | *<LOD* | *<LOD* | 1.66E-06 | 1.69E-06 | 1.79E-06 | 1.96E-06 |
| PFTrDA | *0.01* | *1.51E-11* | *<LOD* | *<LOD* | *<LOD* | *<LOD* | *<LOD* | *<LOD* | *<LOD* | *<LOD* | 3.43E-07 | 3.41E-07 | 3.37E-07 | 3.66E-07 |
| PFTeDA | *0.01* | *1.40E-11* | *<LOD* | *<LOD* | *<LOD* | *<LOD* | *<LOD* | *<LOD* | *<LOD* | *<LOD* | 4.79E-07 | 4.95E-07 | 3.91E-07 | 4.31E-07 |
| PFHxDA | *0.01* | *1.40E-11* | *<LOD* | *<LOD^2^* | *<LOD* | *<LOD* | *<LOD* | *<LOD* | *<LOD* | *<LOD* | *<LOD* | *<LOD* | 6.29E-08 | 7.06E-08 |
| 4:2 FTS | *0.01* | *3.05E-11* | 7.85E-06 | 1.70E-05 | 7.22E-06 | 7.40E-06 | *<LOD* | *<LOD* | 1.61E-06 | 1.46E-05 | 6.69E-06 | 1.49E-06 | 2.46E-08 | 2.31E-08 |
| 6:2 FTS | *0.01* | *2.34E-11* | 2.11E-04 | 4.33E-04 | *<LOD* | *<LOD* | *<LOD* | *<LOD* | 5.82E-05 | 4.19E-04 | *<LOD* | *<LOD* | *<LOD* | *<LOD* |
| 8:2 FTS | *0.02* | *3.80E-11* | 5.03E-05 | 9.52E-05 | 5.07E-05 | *<LOD* | *<LOD* | *<LOD* | 4.29E-05 | 2.09E-04 | *<LOD* | *<LOD* | 8.72E-07 | 1.26E-06 |
| 10:2 FTS | *0.02* | *3.18E-11* | *<LOD* | 6.02E-07 | 5.69E-07 | 6.02E-07 | *<LOD* | *<LOD* | *<LOD* | *<LOD* | 3.84E-06 | 2.10E-06 | 1.63E-08 | 3.13E-08 |

***Note:*** *LOD, Limit of Detection; ppb, parts per billion, µg/L; M, Mol, mol/L*

Table S4: Optimisation on the TOP assay to test AFFF samples, for Figure 5

| TOP assay parameters | | Oxidative indicator  *∑FTS (nM)* | | | |
| --- | --- | --- | --- | --- | --- |
| *K_2_S_2_O_8_, NaOH* | *Steps / T / t* | *#1* | *#2* | *#3* | *#4* |
| 1× ^a^, 8× ^b^ | 1 / 85ºC / 6 hrs | 6.46 | 0.561 | 962 | 1560 |
| 5×, 9× | 1 / 85ºC / 6 hrs | *<LOD* | *<LOD* | 74.8 | 1800 |
| 8×, 10× | 1 / 90ºC / 7 hrs | *<LOD* | *<LOD* | 0.210 | 2.98 |
| s8×, 10× | 2 / 90ºC / 12 hrs | *<LOD* | *<LOD* | 0.182 | 2.83 |

***Note:*** ^a^*1 ×= 0.058 M, and ^b^1 × = ⁓ 0.15 M of each, according to Houtz and Sedlak (2012) (the initial TOP assay, without optimisation). Here, ‘LOD’ indicates the limit of detection (0.0305 mM, 0.0234 nM, 0.0380 nM and 0.0318 nM for 4:2, 6:2, 8:2 and 10:2 FTS, respectively), ‘T’ for temperature and ‘t’ for time, respectively.*

Table S5: Physical and chemical characterisation of soil

| Parameters | Value |
| --- | --- |
| pH-water (1:5) | 7.74 |
| pH-CaCl_2_ (1:5) | 6.8 |
| Conductivity (µS / cm) | 0.063 |
| Moisture content (%) | <0.1 |
| Total carbon (%) | 4.2 |
| Total organic carbon (%) | 3.6 |
| Total organic matter (%) | 6.2 |
| Nitrogen (%) | 2.93 |
| Carbon (%) | 1.26 |
| Total inorganic carbon (%) | 0.016 |

Table S6: Concentrations of individual PFAS compounds in PFAS-contaminated soil, for Figure 6 (all the data below were tabled for the stock samples, after being converted to the initial ones by multiplying the respective dilution factors)

|  | ***LOD*** | | ***Pre TOP assay [Concentration (nM)]*** | | | ***Post TOP assay [Concentration (nM)]*** | | |
| --- | --- | --- | --- | --- | --- | --- | --- | --- |
| ***Detected compounds*** | ***ppb (μg/L)*** | ***nM*** | ***10×*** | ***50×*** | ***100×*** | ***10×*** | ***50×*** | ***100×*** |
| PFBS | *0.01* | *0.0334* | < *LOD* | *LOD* | *LOD* | *LOD* | *LOD* | *LOD* |
| PFHxS | *0.01* | *0.0250* | 34.2 | 43.1 | 121 | 69.4 | 34.1 | 68.7 |
| PFHpS | *0.01* | *0.0222* | *<LOD* | *<LOD* | *<LOD* | 2.73 | 18.8 | 70.9 |
| PFOS | *0.01* | *0.0200* | 504 | 94.4 | 1024 | 852 | 2610 | 14200 |
| PFNS | *0.02* | *0.0400* | 1.24 | *<LOD* | *<LOD* | 3.04 | *<LOD* | *<LOD* |
| PFBA | *0.05* | *0.234* | 76.5 | 62.6 | 73.9 | 174. | 101 | 213 |
| PFPeA | *0.01* | *0.0379* | 85.7 | 73.2 | 101 | 193. | 111 | 162 |
| PFHxA | *0.01* | *0.0318* | 89.1 | 68.8 | 104 | 214 | 121 | 202 |
| PFHpA | *0.01* | *0.0275* | 40.2 | 32.4 | 46.9 | 100 | 223. | 235 |
| PFOA | *0.01* | *0.0242* | 231 | 295 | 476 | 1201 | 1940 | 2680 |
| PFNA | *0.01* | *0.0216* | 31.8 | 14.1 | 38.9 | 59.5 | 35.7 | 75.1 |
| PFDA | *0.01* | *0.0195* | 201 | 27.10 | 263 | 284 | 152 | 402 |
| PFUnDA | *0.01* | *0.0177* | 25.3 | *<LOD* | 34.8 | 23.3 | 11.5 | 46.9 |
| PFDoDA | *0.01* | *0.0163* | *<LOD* | *<LOD* | *<LOD* | *<LOD* | 4.54 | 48.5 |
| PFTrDA | *0.01* | *0.0151* | 8.19 | *<LOD* | 16.9 | 10.8 | *<LOD* | 7.66 |
| PFTeDA | *0.01* | *0.0140* | 2.74 | *<LOD* | 7.75 | 1.76 | 4.89 | 19.3 |
| FOSA | *0.01* | *0.0200* | 7.42 | *<LOD* | 18.9 | 12.7 | 10.6 | 31.6 |
| FOSAA | *0.01* | *0.0174* | 13.9 | *<LOD* | 24.2 | 15.9 | 11.6 | 29.2 |
| EtFOSE | *0.05* | *0.0875* | 2.90 | *<LOD* | *<LOD* | *<LOD* | 4.48 | 11.4 |
| MeFOSAA | *0.02* | *0.0350* | 1.77 | *<LOD* | *<LOD* | 3.09 | *<LOD* | *<LOD* |
| EtFOSAA | *0.01* | *0.0171* | 52.2 | 19.5 | 87.6 | 20.9 | 12.2 | 24.8 |
| MeFOSE | *0.05* | *0.0898* | *<LOD* | *<LOD* | *<LOD* | *<LOD* | 52.5 | 157 |
| 4:2 FTS | *0.01* | *0.0305* | *<LOD* | *<LOD* | *<LOD* | *<LOD* | *<LOD* | *<LOD* |
| 6:2 FTS | *0.01* | *0.0234* | 90.8 | 101 | 273 | 81.1 | *<LOD* | *<LOD* |
| 8:2 FTS | *0.02* | *0.0380* | 18.2 | 25.3 | 39.9 | 12.9 | 6.25 | 12.4 |
| 10:2 FTS | *0.02* | *0.0318* | *<LOD* | *<LOD* | *<LOD* | *<LOD* | *<LOD* | *<LOD* |

***Note:*** *LOD, Limit of Detection; ppb, parts per billion, µg/L; nM, NanoM, nanomol/L*

***R*ecovery Analysis:**

Surrogate recovery in the tested samples was calculated using the following equation (Dasu et al., 2017).

Recovery (%) = $\frac{S_{ar}\times\frac{I_{cs}}{I_{ar}}}{S_{cs}}\times100$ ………………….. ………… Eq. S1

Where *S_ar_* is area of each surrogate standard in the extract; *I_ar_* is area of respective internal standard in the extract; *S_cs_* is area of each surrogate standard in the calibration solution; and *I_cs_* is area of respective internal standard in the calibration solution.

**References**

Dasu, K., Nakayama, S.F., Yoshikane, M., Mills, M.A., Wright, J.M., Ehrlich, S., 2017. An ultra-sensitive method for the analysis of perfluorinated alkyl acids in drinking water using a column switching high-performance liquid chromatography tandem mass spectrometry. Journal of Chromatography A 1494, 46-54.

Houtz, E.F., Sedlak, D.L., 2012. Oxidative conversion as a means of detecting precursors to perfluoroalkyl acids in urban runoff. Environmental science & technology 46, 9342-9349.
